# Health improvement for disadvantaged people in Nepal – an evaluation

**DOI:** 10.1186/1472-698X-12-20

**Published:** 2012-09-26

**Authors:** Ram B Rana, Rabindra Ghimire, Mahendra B Shah, Tirtha Kumal, Elise Whitley, Ian A Baker

**Affiliations:** 1 Local Initiatives for Biodiversity, Research, Development (LI-BIRD), PO Box 324, Pokhara, Kaski, Nepal; 2 The Britain-Nepal Medical Trust, PO Box 20564, Lazimpat, Kathmandu, Nepal; 3 Department of Social Medicine, University of Bristol, Canynge Hall, 30 Whatley Road, Bristol, BS8 2PS, UK

## Abstract

**Background:**

An evaluation of progress with participatory approaches for improvement of health knowledge and health experiences of disadvantaged people in eight Districts of Eastern Nepal has been undertaken.

**Methods:**

A random selection of Village Development Committees and households, within the eight Districts where participation and a Rights-based Approach had been promoted specifically by local NGOs were compared with similar villages and households in eight Districts where this approach had not been promoted. Information was sought by structured interview and observation by experienced enumerators from both groups of householders. Health knowledge and experiences were compared between the two sets of households. Adjustments were made for demographic confounders.

**Results:**

Complete data sets were available for 628 of the 640 households. Health knowledge and experiences were low for both sets of households. However, health knowledge and experiences were greater in the participatory households compared with the non-participatory households. These differences remained after adjustment for confounders.

**Conclusions:**

The study was designed to evaluate progress with participatory processes delivered by non-governmental organisations over a five year period. Improvements in health knowledge and experiences of disadvantaged people were demonstrated in a consistent and robust manner where interventions had taken place.

## Background

A Rights-Based Approach (RBA) for health improvement was introduced for Disadvantaged Groups (DAGs) in 107 villages in 8 Districts through a participatory process of social mobilisation and empowerment by staff of The Britain-Nepal Medical Trust (BNMT) and local NGO partners [1]. Participatory analysis and planning by spouses and by women separately, child -to-child health promotion in schools, street theatre by local groups and mobilisation of youths for health improvement were encouraged and supported [2,3]. Local health committees were revitalised with inclusive representation. Health workers in local health institutions were instructed in non-discriminatory health services. DAG groups were introduced to the concept of health rights. Advocacy for their health rights and improved health status and health care was activated at village, regional and national levels.

In 2009, The Trust sought to evaluate progress of these interventions in terms of improvement of health knowledge/experiences of DAGs in RBA intervention villages compared with adjacent non-RBA intervention villages, as control settings.

## Methods

**Figure 1:**
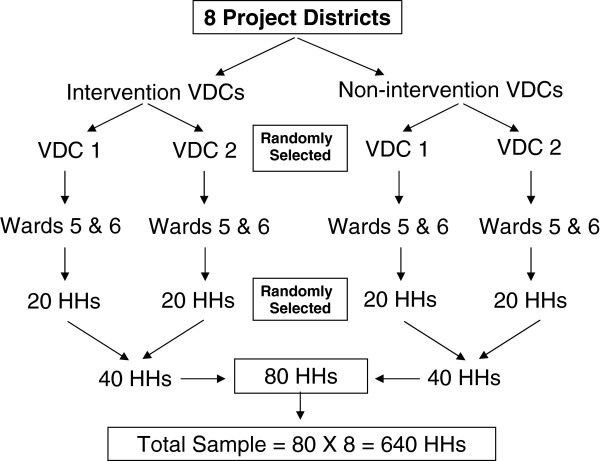
Selection of households(HHs) for interviews.

Villages (Village Development Committees, VDCs) were selected randomly in Districts where participatory processes had been promoted and not promoted. Wards 5&6, which are sub-sections of villages, were identified in each selected VDC. Households (HHs) in these wards were identified by social mapping of all households and 20 households were randomly selected (Figure 1).

320 RBA HHs and 320 non-RBA HHs were invited to interview by village Female Community Health Volunteers (FCHVs) and introduced to enumerators who had experience of a previous 2003–04 survey and a similar range of structured questions. Informal discussion identified the HH decision-maker for interview. Permission to interview was sought and villagers were advised that information they gave would be confidential and anonymised [4].

Knowledge/experience in RBA vs. non-RBA HHs were compared using logistic regression for binary variables. Results are presented as totals and percentages, Odds Ratios (ORs) and 95% Confidence Intervals (CIs) comparing responses from RBA vs. non-RBA HHs. Preliminary analyses suggested that RBA and non-RBA HHs differed in terms of caste, gender, education, food sufficiency, marital status, birth in past 3 years and number of children (Table 1) and analyses were therefore adjusted for these factors. All analyses were carried out using Stata v11.0.

**Table 1:** Frequencies of confounding variables in 628 participants with complete data

**Variable**	**Non-RBA**	**RBA**	**Total**
	**No. (%)**	**No. (%)**	**No. (%)**
Caste			
Brahmin/Chetri	110 (35.4)	58 (18.3)	168 (26.8)
Other	201 (64.6)	259 (81.7)	460 (73.3)
Gender			
Male	117 (37.6)	95 (30.0)	212 (33.8)
Female	194 (62.4)	222 (70.0)	416 (66.2)
Education			
Illiterate	122 (39.2)	105 (33.1)	227 (36.2)
Literate	189 (60.8)	212 (66.9)	401 (63.9)
Food sufficiency			
<9 months	160 (51.5)	176 (55.5)	336 (53.5)
9+ months	151 (48.6)	141 (44.5)	292 (46.5)
Marital status			
Married and together	239 (76.9)	252 (79.5)	491 (78.2)
Other	72 (23.2)	65 (20.5)	137 (21.8)
Birth in last 3 years			
No	235 (75.6)	212 (66.9)	447 (71.2)
Yes	76 (24.4)	105 (33.1)	181 (28.8)
Number of children			
None	51 (16.4)	36 (11.4)	87 (13.9)
One or more	260 (83.6)	281 (88.6)	541 (86.2)

Analyses were based on 628 participants with complete data for variables of interest and all potential confounding variables. Some additional restrictions were used where groups were aware of particular health knowledge.

The evaluation was undertaken as health understanding of common infections, toileting and water sources, reproductive health and family planning and approach to health institutions, health workers and health services. The interviews for the evaluation were detailed and the data was analysed by many components under the above headings. The main tables and illustrative tables are cited in the text. For detailed analyses of all evaluative components, see the link to Additional file 1.

## Results

There were differences in frequency of important characteristics acting as confounders between non-RBA HHs and RBA HHs (Table 1). There are caste differences between the non-RBA and RBA groups in that the non-RBA group has a higher proportion of Brahims and Chetris (35.4% cf. 18.3%). Other differences between the groups are smaller, but with the RBA group having more females, being more literate (66.9% to 60.8%), having less food security (55.5% to 51.5%) and experiencing a birth in the past 3 years more frequently (33.1% to 24.4%). Hence, in further analysis there are multiple adjustments for these differences between the non-RBA and RBA groups.

### Evaluation of health knowledge/experiences of infections

In general, RBA groups held greater knowledge of infections of tuberculosis, HIV and sexually transmitted diseases. Both RBA and non-RBA groups had good understanding of diarrhoeal diseases; pneumonia and malaria, but non-RBA groups had lower responses in terms of care.

Tuberculosis (TB) remains a common infectious illness in Nepal and TB control is exercised by a National Tuberculosis Programme, to which The Britain-Nepal Medical Trust contributes. Hence understanding this disease in terms of symptoms, transmission, prevention and treatment is important. In this study, 568 HHs had heard about TB, with more HHs in the RBA groups (96.3%) being aware of the disease than those in non-RBA HHs (81.3%) Table 2. After multiple adjustments for confounders, the Odds Ratios (ORs) for the RBA group knowledge was 1: 8.95 (95% CIs: 4.48 to 17.88) or almost 9 times more knowledgeable than the non-RBA group. With this expression of TB awareness in 568 HHs, those in non-RBA groups were less aware of symptoms, transmission, prevention and freely available treatment than RBA groups, see Additional file 1: Table 2a-d.

**Table 2:** Odds Ratio (95% CIs) of Knowledge of Tuberculosis

	**N (%) knowledge / no knowledge**	**Unadjusted**	**Multiply adjusted**^**1**^
Intervention group			
Non-RBA	260 (81.3) / 60	1.00	1.00
RBA	308 (96.3) / 12	5.92 (3.11, 11.25))	8.95 (4.48, 17.88)
*P value*		*<0.001*	*<0.001*

^1^ For caste, gender, education, food sufficiency, marital status, birth in last 3 years, number of children in household.

Infection with HIV is much less common in Nepal, but promoting awareness of the disease, its pattern of transmission and means of prevention were important to the RBA process. For those who had heard of HIV infection in this survey (463), information by radio was most common (above 77% in both groups). RBA HHs were additionally more informed by friends and by health workers. Specific knowledge of transmission and prevention of HIV was less common in non-RBA groups compared with RBA groups see Additional file 1 Table 3a-c.

Understanding of other sexually transmitted diseases (STDs) were understood less in non-RBA groups than RBA groups for those who knew of STDs (298) (83.3% : 93.8%, ORs 1:5.86, 95%CIs 2.24 – 15.36) but the overall understanding in all households was less than 50%, see Additional file 1 Table 4a-d.

HHs were surveyed for prevention and treatment of diarrhoea. Table 3 illustrates the components of prevention examined by HH interviews and the advantage to RBA households of their greater knowledge.

**Table 3:** Odds ratio (95% confidence interval) for knowledge of diarrhoea prevention amongst all 628 participants

	**N (%) yes / no**	**Unadjusted**	**Multiply adjusted**^**1**^
Wash hands before eating			
Non-RBA	98 (31.5) / 213	1.00	1.00
RBA	171 (53.9) / 146	2.55 (1.84, 3.53)	2.51 (1.79, 3.53)
*P value*		*<0.001*	*<0.001*
Wash hands with soap after toilet			
Non-RBA	67 (21.5) / 244	1.00	1.00
RBA	158 (49.8) / 159	3.62 (2.55, 5.13)	3.76 (2.60, 5.42)
*P value*		*<0.001*	*<0.001*
Drink clean water			
Non-RBA	152 (48.9) / 159	1.00	1.00
RBA	217 (68.5) / 100	2.27 (1.64, 3.14)	2.23 (1.59, 3.14)
*P value*		*<0.001*	*<0.001*
Don’t eat stale food			
Non-RBA	190 (61.1) / 121	1.00	1.00
RBA	204 (64.4) / 113	1.15 (0.83, 1.59)	1.25 (0.89, 1.76)
*P value*		*0.40*	*0.19*
Cover food			
Non-RBA	63 (20.3) / 248	1.00	1.00
RBA	113 (35.7) / 204	2.18 (1.52, 3.12)	2.11 (1.44, 3.09)
*P value*		*<0.001*	*<0.001*
Don’t overeat			
Non-RBA	55 (17.7) / 256	1.00	1.00
RBA	64 (20.2) / 253	1.18 (0.79, 1.76)	1.03 (0.68, 1.56)
*P value*		*0.42*	*0.90*
	N (%) no / yes^2^	Unadjusted	Multiply adjusted^1^
Don’t know how to prevent			
Non-RBA	285 (91.6) / 26	1.00	1.00
RBA	303 (95.6) / 14	1.97 (1.01, 3.86)	2.13 (1.03, 4.41)
*P value*		*0.05*	*0.04*

^1^ For caste, gender, education, food sufficiency, marital status, birth in last 3 years, number of children in household; ^2^ Outcome is “positive” i.e. negative response to “Don’t know how to prevent”.

RBA groups had a better understanding of water hygiene for prevention of diarrhoea and for drinking clean water. Treatment with oral rehydration solution, navajeevan and jeevan jal, was well understood by both groups as were sources of provision. See Additional file 1 Table 5a-d

Knowledge of the symptoms of pneumonia was well understood in both non-RBA and RBA groups, with RBA groups having greater understanding of specific symptoms. See Additional file 1 Table 6a-d.

Both groups, who had heard of malaria (477), appreciated knowledge of the symptoms of the disease (91.4% : 95.7%) see Additional file 1 Tables 7a-d. There was also good knowledge of transmission of the disease, sources of treatment and means of prevention in both RBA and non-RBA groups. The regular use of nets in HHs was reported as less in non-RBA groups than RBA groups Table 4.

**Table 4:** Odds ratio (95% confidence interval) for use of mosquito net amongst 477 participants who had heard of malaria

	**N (%) yes / no**	**Unadjusted**	**Multiply adjusted**^**1**^
Use mosquito net regularly at home (missing for 4 participants)			
Non-RBA	172 (78.9) / 46	1.00	1.00
RBA	241 (94.5) / 14	4.60 (2.45, 8.64)	4.60 (2.39, 8.86)
*P value*		*<0.001*	*<0.001*

^1^ For caste, gender, education, food sufficiency, marital status, birth in last 3 years, number of children in household.

### Toilets, waste & water

Toilets at the house were less common in non-RBA groups than RBA groups ( 58.4% : 65.3%, OR 1:2.02, 95% CIs 1.28 – 3.18) but the percentages in each were not high. A quarter of both groups used waste land (khet bari) and other locations for toileting.

Waste materials were less likely to be disposed of in a pit or burnt in non-RBA groups and these groups tended to dispose of rubbish on waste land (bari), (65.9% : 48.0%, OR 1: 0.44, 95%CIs 0.31 – 0.62). Water was commonly sourced from piped sources or from hand pumps by both groups. See Additional file 1 Tables 8a-c.

### Evaluation of reproductive health & family planning

There was a lower level of knowledge of good reproductive health in terms of the benefits of Ante Natal Care and the course of pregnancy and delivery for both RBA and non-RBA groups, with non-RBA groups at a greater disadvantage. Family Planning was well understood by women especially in both groups. There was a low level of knowledge of the legality of abortion.

In responses to interviews with the HH decision-maker, 416 females replied compared with 212 males. There was a higher% of female responders in the RBA group compared with the non-RBA group (70.0% : 62.4%) For reproductive health, 66.4% of responders overall in the RBA group were aware of the recommended x4 Ante Natal Visits compared with 55.6% in the non-RBA group Table 5. Awareness of these recommended levels was not high in either group.

**Table 5:** Odds ratio (95% confidence interval) for knowledge of antenatal visit frequency amongst all 628 participants

	**N (%) yes / no**	**Unadjusted**	**Multiply adjusted**^**1**^
Antenatal visit 4+ times			
Non-RBA	173 (55.6) / 138	1.00	1.00
RBA	209 (66.4) / 106	1.57 (1.14, 2.17)	1.45 (1.02, 2.05)
*P value*		*0.01*	*0.04*

(2 have missing response for this question).

^1^ For caste, gender, education, food sufficiency, marital status, birth in last 3 years, number of children in household.

Little difference accrued to either group in terms of knowledge of danger signs in pregnancy, but awareness of specific dangers, apart from excessive bleeding, was low.

Knowledge of the Nepalese Birth Preparedness Package (BPP) was known to only 242 HHs, less than 50%. There was little difference in awareness of the BPP between the two groups. No more than 24% had health institutional births. Only 14% had help from a Skilled Birth Attendant and only 55% knew of Clean Birthing Kits.

The need for care of the newborn in general was well understood in both groups, but there were advantages to the RBA group around specific care although these were of low appreciation. Danger signs in the newborn was less understood in the non-RBA group.

For Family Planning (FP), there was little difference in knowledge between the two groups in methods of contraception (96.9% : 97.4% ) or of specific methods in the 602 responding HHs.see Additional file 1 Tables 9a-p. Permanent contraception was well known for 1female tubal ligation/laparoscopy in both groups (79.7% : 87.5%, OR 1:1.81, 95%CIs 1.14 – 2.88) and for vasectomy for the RBA group compared with the non-RBA group(57.4% : 76.5%, OR 1:3.39, 95%CIs 2.28 – 5.05). Sources of temporary contraceptives was known to both groups (95.8% : 97.4%) and in particular these sources centred around health institutions (95.2% : 95.8%).

There is a low level of knowledge of the legality of abortion in these HHs. Non-RBA groups understood legal grounds less than RBA groups (20.9% : 41.3%, OR 1:3.38, 95%CIs 2.24 – 5.09). There was a similar low understanding of the grounds which made abortion illegal (12.9% : 35.7%, OR 1: 4.32, 95%CIs 2.77 – 6.73) see Additional file 1 Tables 9a-p.

### Health institutions, health workers & health rights

Knowing to use the Health Institution, usually a Health Post or Sub-Health Post, when sick was appreciated by both non-RBA and RBA groups (97.1% : 98.7%). But little more than half for RBA groups and less than half for non-RBA groups appreciated functions for health education, family planning, ante-natal care, immunisation and free TB treatment.

Satisfaction with use of the services of the Dhami Jankari (traditional healer) was 61.5% of non-RBA groups and 50.3% of RBA groups (OR 1: 0.63, 95% CIs 0.45 – 0.87). See Additional file 1 Tables 10a-f.

There was limited knowledge of the concept of Health Rights. Only 11.0% in HHs in the non-RBA group and 44.3% of HHs in the RBA group (OR 1:10.5, 95%CIs 6.40 – 17.22) understood their Health Rights, Table 6.

**Table 6:** Odds (95% CI) of Health Rights knowledge

	**N (%) knowledge / no knowledge**	**Unadjusted**	**Adjusted for intervention group**	**Multiply adjusted**
Intervention group				
Non-RBA	35 (11.0) / 284	1.00		1.00
RBA	141 (44.3) / 177	6.46 (4.27, 9.79)		10.50 (6.40, 17.22)
*P value*		*<0.001*		*<0.001*
Caste				
Brahmin/Chhetri	45 (26.8) / 123	1.00	1.00	1.00
Janjati	82 (29.9) / 192	1.17 (0.76, 1.79)	0.73 (0.45, 1.18)	0.78 (0.46, 1.31)
Janjati-terai	1 ( 2.1) / 47	0.06 (0.01, 0.43)	0.03 (0.00, 0.22)	0.02 (0.00, 0.18)
Dalit	36 (30.3) / 83	1.19 (0.71, 1.99)	0.76 (0.42, 1.36)	1.07 (0.54, 2.10)
Others	9 (40.9) / 13	1.89 (0.76, 4.73)	1.94 (0.69, 5.52)	1.68 (0.55, 5.18)
*P value*		*<0.001*	*<0.001*	*<0.001*
Gender				
Female	105 (24.9) / 317	1.00	1.00	1.00
Male	71 (33.0) / 144	1.49 (1.04, 2.13)	1.92 (1.29, 2.87)	1.72 (1.10, 2.70)
*P value*		*0.03*	*0.001*	*0.02*
Education				
Illiterate	42 (18.7) / 183	1.00	1.00	1.00
Literate	43 (29.1) / 105	1.78 (1.10, 2.91)	1.63 (0.96, 2.74)	1.42 (0.81, 2.49)
Lower 2ndary	24 (21.2) / 89	1.17 (0.67, 2.06)	1.16 (0.64, 2.12)	1.12 (0.58, 2.15)
2ndary and above	67 (45.9) / 79	3.70 (2.32, 5.90)	4.21 (2.51, 7.04)	4.09 (2.26, 7.42)
*P value*		*<0.001*	*<0.001*	*<0.001*

## Discussion

The current profile of DAG households in RBA VDCs was dominated by Janajatis (indigenous groups) and Dalits with only 18.3% in the Brahmin/Chettri castes, implying considerable structural inequality. Females most often responded as decision-makers to the interviews (70.3%). These DAG households were at a disadvantage socio-economically, as indicated by possessing less food security, (Table 1).

There was some evidence that randomisation was not entirely successful in producing two comparable groups. Adjacent non-RBA supported VDCs had a 2:1 ratio of female to male decision-makers also, a higher percentage of Brahmins/Chettris (35.4%) but a higher percentages of illiterate (39.2% : 33.1%). Differences between the RBA and non-RBA groups may relate in part to these demographic factors. Hence, multiple adjustments of these factors have been made for the Odds Ratios (ORs) of health knowledge/experiences for non-RBA groups and RBA groups, together with 95% Confidence Intervals. These ORs of the RBA groups are consistent and robust even with the bias of non-RBA groups having nearly double the percentage of higher caste Brahmins and Chettris. Interactions between variables were generally consistent with greater impact of RBA intervention in lower caste, less educated and those having lower food sufficiency. With many comparisons, Type I errors (the incorrect rejection of a true null hypothesis) are likely and the focus of the evaluation has therefore been on the consistency and magnitude of associations rather than on the results of individual significance tests.

Improved Reproductive Health is a future concern for poor communities with high mortalities and morbidities to mothers and babies and participatory changes need further development [5]. Possession of the concept and understanding of Health Rights was also limited [6]. The strength of the interventions contrasts with knowledge and services rendered to non-RBA groups, which were generally limited and passive.

The results have been fed back in workshops with health officers locally in Eastern Nepal and to politicians and health officials of the Nepalese Government as a form of advocacy and comparison with national health programmes.

### Limitations

Randomisation did not provide equal characteristics between intervention and non-intervention Districts. Adjustments were made, which may contribute to differences between households’ health. The odds-ratios were however consistent and robust in the expected direction for interventions. The non-intervention Districts were not known to have received direct health interventions, other than those which applied to all Districts following the Government of Nepal’s health plans [7]. The current evaluation acknowledged difficulties in logistics and geographical terrain, but still achieved worthwhile comparisons.

## Conclusions

This evaluation has demonstrated benefits in health knowledge and health experiences to disadvantaged groups in Nepal, when spouse-pairs, women, youths and children are engaged in participatory approaches by local NGOs and co-ordinated by an international NGO, BNMT. Some health responses in both intervention groups and non-intervention groups need more assistance from health service workers and local NGOs.

## Competing interests

None of the authors had competing interests.

## Authors’ contributions

RBR, RG, MBS and TK were involved in the field survey, data processing, preliminary analysis and early reporting. IAB and EW helped with further analysis and final editing of the paper. The authors made equal contributions and have read the final manuscript.

## Pre-publication history

The pre-publication history for this paper can be accessed here:

http://www.biomedcentral.com/1472-698X/12/20/prepub

## Supplementary Material

Additional file 1 Tuberculosis. Click here for file
