# Supplementary material for: Health improvement for disadvantaged people in Nepal – an evaluation
Source: BMC Int Health Hum Rights. 2012 Sep 26;12:20. doi: 10.1186/1472-698X-12-20 (PMC3489826; doi:10.1186/1472-698X-12-20)
Supplement: Additional file 1 — Tuberculosis. [file 1472-698X-12-20-S1.doc]

**Additional Material Files**

**Additional File: Tuberculosis**

**Table 2**a : Odds ratio (95% confidence interval) for knowledge of specific TB symptoms amongst 556 participants who had heard of TB

|  | N (%) yes / no | Unadjusted | Multiply adjusted^1^ |
| --- | --- | --- | --- |
|  |  |  |  |
| Continuous coughing for more than 2 weeks | | | |
| Non-RBA  RBA | 127 (50.6) / 124  230 (75.4) / 75 | 1.00  2.99 (2.09, 4.29) | 1.00  3.44 (2.33, 5.08) |
| *P value* |  | *<0.001* | *<0.001* |
|  |  |  |  |
| Fever | | | |
| Non-RBA  RBA | 143 (57.0) / 108  205 (67.2) / 100 | 1.00  1.55 (1.10, 2.19) | 1.00  1.67 (1.16, 2.41) |
| *P value* |  | *0.01* | *0.01* |
|  |  |  |  |
| Chest pain | | | |
| Non-RBA  RBA | 69 (27.5) / 182  156 (51.2) / 149 | 1.00  2.76 (1.93, 3.94) | 1.00  3.00 (2.04, 4.39) |
| *P value* |  | *<0.001* | *<0.001* |
|  |  |  |  |
| Blood in sputum | | | |
| Non-RBA  RBA | 145 (57.8) / 106  220 (72.1) / 85 | 1.00  1.89 (1.33, 2.70) | 1.00  1.86 (1.28, 2.69) |
| *P value* |  | *<0.001* | *0.001* |
|  |  |  |  |
| Weight loss | | | |
| Non-RBA  RBA | 67 (26.7) / 184  158 (51.8) / 147 | 1.00  2.95 (2.06, 4.23) | 1.00  3.12 (2.12, 4.57) |
| *P value* |  | *<0.001* | *<0.001* |
|  |  |  |  |
| Loss of appetite | | | |
| Non-RBA  RBA | 35 (13.9) / 216  90 (29.5) / 215 | 1.00  2.58 (1.67, 3.99) | 1.00  2.93 (1.84, 4.67) |
| *P value* |  | *<0.001* | *<0.001* |
|  |  |  |  |
|  | N (%) no / yes^2^ | Unadjusted | Multiply adjusted^1^ |
|  |  |  |  |
| Don’t know any symptoms² | | | |
| Non-RBA  RBA | 210 (83.7) / 41  284 (93.1) / 21 | 1.00  2.64 (1.52, 4.60) | 1.00  2.77 (1.55, 4.96) |
| *P value* |  | *0.001* | *0.001* |
|  |  |  |  |

^1^ For caste, gender, education, food sufficiency, marital status, birth in last 3 years, number of children in household; ^2^ Outcome is “positive” i.e. negative response to “Don’t know any symptoms”

**Table 2b:** Odds ratio (95% confidence interval) for knowledge of free TB treatment amongst 556 participants who had heard of TB

|  | N (%) no / yes^2^ | Unadjusted | Multiply adjusted^1^ |
| --- | --- | --- | --- |
|  |  |  |  |
| Do you need to pay for TB treatment? | | | |
| Non-RBA  RBA | 206 (82.4) / 44  279 (91.8) / 25 | 1.00  2.38 (1.41, 4.02) | 1.00  2.80 (1.61, 4.87) |
| *P value* |  | *0.001* | *<0.001* |
|  |  |  |  |

^1^ For caste, gender, education, food sufficiency, marital status, birth in last 3 years, number of children in household; ^2^ Coded as No (correct) vs. Yes or don’t know

**Table 2c:** Odds ratio (95% confidence interval) for knowledge of how TB spreads amongst 556 participants who had heard of TB

|  | N (%) yes / no | Unadjusted | Multiply adjusted^1^ |
| --- | --- | --- | --- |
|  |  |  |  |
| Coughing and sneezing | | | |
| Non-RBA  RBA | 105 (41.8) / 146  215 (70.5) / 90 | 1.00  3.32 (2.34, 4.72) | 1.00  4.12 (2.78, 6.09) |
| *P value* |  | *<0.001* | *<0.001* |
|  |  |  |  |
| Wearing clothes worn by TB patients | | | |
| Non-RBA  RBA | 73 (29.1) / 178  136 (44.6) / 169 | 1.00  1.96 (1.38, 2.79) | 1.00  2.18 (1.49, 3.18) |
| *P value* |  | *<0.001* | *<0.001* |
|  |  |  |  |
| Using utensils used by TB patient | | | |
| Non-RBA  RBA | 76 (30.3) / 175  131 (43.0) / 174 | 1.00  1.73 (1.22, 2.46) | 1.00  1.72 (1.19, 2.49) |
| *P value* |  | *0.002* | *0.004* |
|  |  |  |  |
| Talking to TB patients | | | |
| Non-RBA  RBA | 49 (19.5) / 202  104 (34.1) / 201 | 1.00  2.13 (1.44, 3.16) | 1.00  2.03 (1.35, 3.06) |
| *P value* |  | *<0.001* | *0.001* |
|  |  |  |  |
| Through the air | | | |
| Non-RBA  RBA | 50 (19.9) / 201  107 (35.1) / 198 | 1.00  2.17 (1.47, 3.21) | 1.00  2.24 (1.48, 3.39) |
| *P value* |  | *<0.001* | *<0.001* |
|  |  |  |  |
| Through flies | | | |
| Non-RBA  RBA | 10 (4.0) / 241  8 (2.6) / 297 | 1.00  0.65 (0.25, 1.67) | 1.00  0.62 (0.23, 1.68) |
| *P value* |  | *0.37* | *0.35* |
|  |  |  |  |
| Sitting together with TB patient | | | |
| Non-RBA  RBA | 70 (27.9) / 181  104 (34.1) / 201 | 1.00  1.34 (0.93, 1.92) | 1.00  1.48 (1.01, 2.19) |
| *P value* |  | *0.12* | *0.05* |
|  |  |  |  |
| Eating from TB patient’s plate | | | |
| Non-RBA  RBA | 117 (46.6) / 134  159 (52.1) / 146 | 1.00  1.25 (0.89, 1.74) | 1.00  1.23 (0.87, 1.75) |
| *P value* |  | *0.20* | *0.24* |
|  |  |  |  |
|  | N (%) no / yes^2^ | Unadjusted | Multiply adjusted^1^ |
|  |  |  |  |
| Don’t know how it spreads | | | |
| Non-RBA  RBA | 198 (78.9) / 53  288 (94.4) / 17 | 1.00  4.53 (2.55, 8.06) | 1.00  4.72 (2.59, 8.60) |
| *P value* |  | *<0.001* | *<0.001* |
|  |  |  |  |

^1^ For caste, gender, education, food sufficiency, marital status, birth in last 3 years, number of children in household; ^2^ Outcome is “positive” i.e. negative response to “Don’t know how it spreads”

**Table 2d:** Odds ratio (95% confidence interval) for knowledge of TB prevention amongst 556 participants who had heard of TB

|  | N (%) yes / no | Unadjusted | Multiply adjusted^1^ |
| --- | --- | --- | --- |
|  |  |  |  |
| Cover mouth and nose while coughing or sneezing | | | |
| Non-RBA  RBA | 83 (33.1) / 168  209 (68.5) / 96 | 1.00  4.41 (3.08, 6.30) | 1.00  4.97 (3.37, 7.33) |
| *P value* |  | *<0.001* | *<0.001* |
|  |  |  |  |
| Not spitting everywhere | | | |
| Non-RBA  RBA | 52 (20.7) / 199  135 (44.3) / 170 | 1.00  3.04 (2.08, 4.44) | 1.00  3.06 (2.05, 4.56) |
| *P value* |  | *<0.001* | *<0.001* |
|  |  |  |  |
| Balanced diet | | | |
| Non-RBA  RBA | 54 (21.5) / 197  122 (40.0) / 183 | 1.00  2.43 (1.67, 3.55) | 1.00  2.12 (1.43, 3.15) |
| *P value* |  | *<0.001* | *<0.001* |
|  |  |  |  |
| Avoid contact with TB patients | | | |
| Non-RBA  RBA | 117 (46.6) / 134  169 (55.4) / 136 | 1.00  1.42 (1.02, 1.99) | 1.00  1.42 (1.00, 2.02) |
| *P value* |  | *0.04* | *0.05* |
|  |  |  |  |
| BCG vaccination | | | |
| Non-RBA  RBA | 11 ( 4.4) / 240  36 (11.8) / 269 | 1.00  2.92 (1.45, 5.96) | 1.00  2.46 (1.20, 5.05) |
| *P value* |  | *0.003* | *0.01* |
|  |  |  |  |
|  | N (%) no / yes^2^ | Unadjusted | Multiply adjusted^1^ |
|  |  |  |  |
| Don’t know how to prevent | | | |
| Non-RBA  RBA | 184 (73.3) / 67  275 (90.2) / 30 | 1.00  3.34 (2.09, 5.34) | 1.00  3.46 (2.11, 5.68) |
| *P value* |  | *<0.001* | *<0.001* |
|  |  |  |  |

^1^ For caste, gender, education, food sufficiency, marital status, birth in last 3 years, number of children in household; ^2^ Outcome is “positive” i.e. negative response to “Don’t know how to prevent”

**Additional File: HIV**

**Table 3a:** Odds ratio (95% confidence interval) for source of HIV knowledge amongst 463 participants who had heard of HIV

|  | N (%) yes / no | Unadjusted | Multiply adjusted^1^ |
| --- | --- | --- | --- |
|  |  |  |  |
| Family | | | |
| Non-RBA  RBA | 22 (11.5) / 169  63 (23.2) / 209 | 1.00  2.32 (1.37, 3.92) | 1.00  2.76 (1.57, 4.86) |
| *P value* |  | *0.002* | *<0.001* |
|  |  |  |  |
| Friends, neighbours and relatives | | | |
| Non-RBA  RBA | 90 (47.1) / 101  170 (62.5) / 102 | 1.00  1.87 (1.28, 2.72) | 1.00  1.75 (1.17, 2.61) |
| *P value* |  | *0.001* | *0.01* |
|  |  |  |  |
| Health workers | | | |
| Non-RBA  RBA | 57 (29.8) / 134  154 (56.6) / 118 | 1.00  3.07 (2.07, 4.54) | 1.00  3.06 (2.03, 4.63) |
| *P value* |  | *<0.001* | *<0.001* |
|  |  |  |  |
| Teacher, school and students | | | |
| Non-RBA  RBA | 24 (12.6) / 167  55 (20.2) / 217 | 1.00  1.76 (1.05, 2.97) | 1.00  1.95 (1.12, 3.39) |
| *P value* |  | *0.03* | *0.02* |
|  |  |  |  |
| TV | | | |
| Non-RBA  RBA | 59 (30.9) / 132  52 (19.1) / 220 | 1.00  0.53 (0.34, 0.81) | 1.00  0.63 (0.40, 0.99) |
| *P value* |  | *0.004* | *0.05* |
|  |  |  |  |
| Radio | | | |
| Non-RBA  RBA | 150 (78.5) / 41  212 (77.9) / 60 | 1.00  0.97 (0.62, 1.51) | 1.00  1.31 (0.80, 2.13) |
| *P value* |  | *0.88* | *0.28* |
|  |  |  |  |
| Books and newspapers | | | |
| Non-RBA  RBA | 36 (18.9) / 155  57 (21.0) / 215 | 1.00  1.14 (0.72, 1.82) | 1.00  1.38 (0.83, 2.29) |
| *P value* |  | *0.58* | *0.22* |
|  |  |  |  |
| Hoarding boards | | | |
| Non-RBA  RBA | 6 (3.1) / 185  16 (5.9) / 256 | 1.00  1.93 (0.74, 5.02) | 1.00  2.30 (0.84, 6.30) |
| *P value* |  | *0.18* | *0.11* |
|  |  |  |  |
| Cinema hall | | | |
| Non-RBA  RBA | 3 (1.6) / 188  3 (1.1) / 269 | 1.00  0.70 (0.14, 3.50) | 1.00  0.94 (0.16, 5.45) |
| *P value* |  | *0.66* | *0.95* |
|  |  |  |  |
| Poster, pamphlet and booklets | | | |
| Non-RBA  RBA | 11 ( 5.8) / 180  33 (12.1) / 239 | 1.00  2.26 (1.11, 4.59) | 1.00  2.09 (0.99, 4.41) |
| *P value* |  | *0.02* | *0.05* |
|  |  |  |  |
|  | N (%) no / yes^2^ | Unadjusted | Multiply adjusted^1^ |
|  |  |  |  |
| Don’t know source | | | |
| Non-RBA  RBA | 189 (99.0) / 2  271 (99.6) / 1 | 1.00  2.87 (0.26, 31.85) | 1.00  4.07 (0.29, 57.32) |
| *P value* |  | *0.39* | *0.30* |
|  |  |  |  |

^1^ For caste, gender, education, food sufficiency, marital status, birth in last 3 years, number of children in household; ^2^ Outcome is “positive” i.e. negative response to “Don’t know source”

**Table 3b:** **Odds ratio (95% confidence interval) for HIV transmission knowledge amongst 463 participants who had heard of HIV**

|  | N (%) yes / no | Unadjusted | Multiply adjusted^1^ |
| --- | --- | --- | --- |
|  |  |  |  |
| Unsafe sex | | | |
| Non-RBA  RBA | 140 (73.3) / 51  249 (91.5) / 23 | 1.00  3.94 (2.31, 6.73) | 1.00  4.25 (2.41, 7.48) |
| *P value* |  | *<0.001* | *<0.001* |
|  |  |  |  |
| Use of unsterilised syringe | | | |
| Non-RBA  RBA | 66 (34.6) / 125  133 (48.9) / 139 | 1.00  1.81 (1.24, 2.65) | 1.00  1.97 (1.31, 2.98) |
| *P value* |  | *0.002* | *0.001* |
|  |  |  |  |
| Exchanging needles/syringes | | | |
| Non-RBA  RBA | 35 (18.3) / 156  120 (44.1) / 152 | 1.00  3.52 (2.27, 5.45) | 1.00  3.57 (2.25, 5.66) |
| *P value* |  | *<0.001* | *<0.001* |
|  |  |  |  |
| Contaminated blood | | | |
| Non-RBA  RBA | 54 (28.3) / 137  137 (50.4) / 135 | 1.00  2.57 (1.74, 3.82) | 1.00  2.57 (1.68, 3.93) |
| *P value* |  | *<0.001* | *<0.001* |
|  |  |  |  |
| Infected mother to baby | | | |
| Non-RBA  RBA | 18 ( 9.4) / 173  86 (31.6) / 186 | 1.00  4.44 (2.57, 7.69) | 1.00  4.37 (2.46, 7.77) |
| *P value* |  | *<0.001* | *<0.001* |
|  |  |  |  |
| Shaking hands with HIV infected person | | | |
| Non-RBA  RBA | 4 (2.1) / 187  7 (2.6) / 365 | 1.00  1.23 (0.36, 4.28) | 1.00  1.24 (0.33, 4.67) |
| *P value* |  | *0.74* | *0.75* |
|  |  |  |  |
| Sitting together with HIV infected person | | | |
| Non-RBA  RBA | 10 (5.2) / 181  10 (3.7) / 262 | 1.00  0.69 (0.28, 1.69) | 1.00  0.94 (0.36, 2.45) |
| *P value* |  | *0.42* | *0.90* |
|  |  |  |  |
| Sharing meal with HIV infected person | | | |
| Non-RBA  RBA | 14 (7.3) / 177  22 (8.1) / 250 | 1.00  1.11 (0.55, 2.23) | 1.00  1.32 (0.63, 2.78) |
| *P value* |  | *0.76* | *0.46* |
|  |  |  |  |
| Mosquito bite | | | |
| Non-RBA  RBA | 5 (2.6) / 186  9 (3.3) / 263 | 1.00  1.27 (0.42, 3.86) | 1.00  1.39 (0.43, 4.51) |
| *P value* |  | *0.67* | *0.59* |
|  |  |  |  |
|  | N (%) no / yes^2^ | Unadjusted | Multiply adjusted^1^ |
|  |  |  |  |
| Don’t know how HIV is transmitted | | | |
| Non-RBA  RBA | 147 (77.0) / 44  249 (91.5) / 23 | 1.00  3.24 (1.88, 5.58) | 1.00  3.46 (1.95, 6.16) |
| *P value* |  | *<0.001* | *<0.001* |
|  |  |  |  |

^1^ For caste, gender, education, food sufficiency, marital status, birth in last 3 years, number of children in household; ^2^ Outcome is “positive” i.e. negative response to “Don’t know how HIV is transmitted”

**Table 3c**: Odds ratio (95% confidence interval) for HIV prevention knowledge amongst 463 participants who had heard of HIV

|  | N (%) yes / no | Unadjusted | Multiply adjusted^1^ |
| --- | --- | --- | --- |
|  |  |  |  |
| Not having sex with HIV infected person | | | |
| Non-RBA  RBA | 124 (64.9) / 67  240 (88.2) / 32 | 1.00  4.05 (2.52, 6.51) | 1.00  4.45 (2.67, 7.39) |
| *P value* |  | *<0.001* | *<0.001* |
|  |  |  |  |
| Having sex with trusted single partner | | | |
| Non-RBA  RBA | 42 (22.0) / 149  130 (47.8) / 142 | 1.00  3.25 (2.14, 4.93) | 1.00  3.49 (2.24, 5.43) |
| *P value* |  | *<0.001* | *<0.001* |
|  |  |  |  |
| Using condom while having sex | | | |
| Non-RBA  RBA | 65 (34.0) / 126  131 (48.2) / 141 | 1.00  1.80 (1.23, 2.64) | 1.00  1.87 (1.25, 2.82) |
| *P value* |  | *0.003* | *0.003* |
|  |  |  |  |
| Using sterilised syringe only | | | |
| Non-RBA  RBA | 39 (20.4) / 152  79 (29.0) / 193 | 1.00  1.60 (1.03, 2.47) | 1.00  1.54 (0.95, 2.48) |
| *P value* |  | *0.04* | *0.08* |
|  |  |  |  |
| Use safe blood | | | |
| Non-RBA  RBA | 33 (17.3) / 158  74 (27.2) / 198 | 1.00  1.79 (1.13, 2.84) | 1.00  1.66 (1.02, 2.71) |
| *P value* |  | *0.01* | *0.04* |
|  |  |  |  |
| Don’t mix up with HIV infected person | | | |
| Non-RBA  RBA | 7 (3.7) / 184  18 (6.6) / 254 | 1.00  1.86 (0.76, 4.55) | 1.00  1.93 (0.75, 4.93) |
| *P value* |  | *0.17* | *0.17* |
|  |  |  |  |
|  | N (%) no / yes^2^ | Unadjusted | Multiply adjusted^1^ |
|  |  |  |  |
| Don’t know how to prevent HIV | | | |
| Non-RBA  RBA | 141 (73.8) / 50  249 (91.5) / 23 | 1.00  3.84 (2.25, 6.56) | 1.00  4.22 (2.38, 7.50) |
| *P value* |  | *<0.001* | *<0.001* |
|  |  |  |  |

^1^ For caste, gender, education, food sufficiency, marital status, birth in last 3 years, number of children in household; ^2^ Outcome is “positive” i.e. negative response to “Don’t know how to prevent HIV”

**Additional File: Sexually Transmitted Diseases**

**Table 4a: Odds ratio (95% confidence interval) for knowledge of STD transmission amongst 298 participants who had heard of STD**s

|  | N (%) yes / no | Unadjusted | Multiply adjusted^1^ |
| --- | --- | --- | --- |
|  |  |  |  |
| Unsafe sex | | | |
| Non-RBA  RBA | 73 (81.1) / 17  193 (92.8) / 15 | 1.00  3.00 (1.42, 6.31) | 1.00  5.25 (2.11, 13.05) |
| *P value* |  | *0.004* | *<0.001* |
|  |  |  |  |
| Using unsterilised needles | | | |
| Non-RBA  RBA | 34 (37.8) / 56  83 (39.9) / 125 | 1.00  1.09 (0.66, 1.82) | 1.00  1.08 (0.62, 1.88) |
| *P value* |  | *0.73* | *0.79* |
|  |  |  |  |
| Contaminated blood | | | |
| Non-RBA  RBA | 31 (34.4) / 59  103 (49.5) / 105 | 1.00  1.87 (1.12, 3.12) | 1.00  1.72 (0.97, 3.03) |
| *P value* |  | *0.02* | *0.06* |
|  |  |  |  |
| Shaking hands with infected person | | | |
| Non-RBA  RBA | 7 (7.8) / 83  8 (3.9) / 200 | 1.00  0.47 (0.17, 1.35) | 1.00  0.59 (0.18, 1.95) |
| *P value* |  | *0.16* | *0.39* |
|  |  |  |  |
| Sitting together with infected person | | | |
| Non-RBA  RBA | 5 (5.6) / 85  6 (2.9) / 202 | 1.00  0.50 (0.15, 1.70) | 1.00  0.84 (0.21, 3.27) |
| *P value* |  | *0.27* | *0.80* |
|  |  |  |  |
| Sharing meal with infected person | | | |
| Non-RBA  RBA | 13 (14.4) / 77  10 ( 4.8) / 198 | 1.00  0.30 (0.13, 0.71) | 1.00  0.51 (0.19, 1.35) |
| *P value* |  | *0.01* | *0.17* |
|  |  |  |  |
|  | N (%) no / yes^2^ | Unadjusted | Multiply adjusted^1^ |
|  |  |  |  |
| Don’t know about transmission | | | |
| Non-RBA  RBA | 75 (83.3) / 15  195 (93.8) / 13 | 1.00  3.00 (1.36, 6.60) | 1.00  5.86 (2.24, 15.36) |
| *P value* |  | *0.01* | *<0.001* |
|  |  |  |  |

^1^ For caste, gender, education, food sufficiency, marital status, birth in last 3 years, number of children in household; ^2^ Outcome is “positive” i.e. negative response to “Don’t know about transmission”

**Table 4b: Odds ratio (95% confidence interval) for knowledge of STD prevention amongst 298 participants who had heard of STD**s

|  | N (%) yes / no | Unadjusted | Multiply adjusted^1^ |
| --- | --- | --- | --- |
|  |  |  |  |
| Not having sex with infected person | | | |
| Non-RBA  RBA | 68 (75.6) / 22  184 (88.5) / 24 | 1.00  2.48 (1.31, 4.71) | 1.00  3.67 (1.75, 7.71) |
| *P value* |  | *0.01* | *0.001* |
|  |  |  |  |
| No sex with multiple partners | | | |
| Non-RBA  RBA | 34 (37.8) / 56  130 (62.5) / 78 | 1.00  2.75 (1.65, 4.57) | 1.00  2.68 (1.53, 4.69) |
| *P value* |  | *<0.001* | *0.001* |
|  |  |  |  |
| Use of condom | | | |
| Non-RBA  RBA | 34 (37.8) / 56  108 (51.9) / 100 | 1.00  1.78 (1.07, 2.95) | 1.00  1.82 (1.04, 3.20) |
| *P value* |  | *0.03* | *0.04* |
|  |  |  |  |
| Use sterilised needle | | | |
| Non-RBA  RBA | 17 (18.9) / 73  31 (14.9) / 177 | 1.00  0.75 (0.39, 1.44) | 1.00  0.56 (0.27, 1.15) |
| *P value* |  | *0.39* | *0.11* |
|  |  |  |  |
| Use of safe blood | | | |
| Non-RBA  RBA | 8 ( 8.9) / 82  26 (12.5) / 182 | 1.00  1.46 (0.64, 3.37) | 1.00  1.32 (0.52, 3.33) |
| *P value* |  | *0.37* | *0.55* |
|  |  |  |  |
| No mixing with infected person | | | |
| Non-RBA  RBA | 8 (8.9) / 82  10 (4.8) / 198 | 1.00  0.52 (0.20, 1.36) | 1.00  0.74 (0.25, 2.15) |
| *P value* |  | *0.18* | *0.58* |
|  |  |  |  |
|  | N (%) no / yes^2^ | Unadjusted | Multiply adjusted^1^ |
|  |  |  |  |
| Don’t know about prevention | | | |
| Non-RBA  RBA | 74 (82.2) / 16  193 (92.8) / 15 | 1.00  2.78 (1.31, 5.91) | 1.00  5.94 (2.39, 14.79) |
| *P value* |  | *0.01* | *<0.001* |
|  |  |  |  |

^1^ For caste, gender, education, food sufficiency, marital status, birth in last 3 years, number of children in household; ^2^ Outcome is “positive” i.e. negative response to “Don’t know about prevention”

**Table 4c: Odds ratio (95% confidence interval) for knowledge of STD symptoms amongst 298 participants who had heard of STD**

|  | N (%) yes / no | Unadjusted | Multiply adjusted^1^ |
| --- | --- | --- | --- |
|  |  |  |  |
| Pain or burning sensation while peeing | | | |
| Non-RBA  RBA | 54 (60.0) / 36  153 (73.6) / 55 | 1.00  1.85 (1.10, 3.13) | 1.00  2.05 (1.15, 3.68) |
| *P value* |  | *0.02* | *0.02* |
|  |  |  |  |
| Lower abdominal pain | | | |
| Non-RBA  RBA | 46 (51.1) / 44  128 (61.5) / 80 | 1.00  1.53 (0.93, 2.52) | 1.00  1.56 (0.90, 2.70) |
| *P value* |  | *0.10* | *0.12* |
|  |  |  |  |
| Lesions around genitals | | | |
| Non-RBA  RBA | 41 (45.6) / 49  136 (65.4) / 72 | 1.00  2.26 (1.36, 3.74) | 1.00  1.96 (1.14, 3.39) |
| *P value* |  | *0.002* | *0.02* |
|  |  |  |  |
| Swelling of lymph nodes | | | |
| Non-RBA  RBA | 10 (11.1) / 80  43 (20.7) / 165 | 1.00  2.08 (1.00, 4.36) | 1.00  2.33 (1.05, 5.18) |
| *P value* |  | *0.05* | *0.04* |
|  |  |  |  |
|  | N (%) no / yes^2^ | Unadjusted | Multiply adjusted^1^ |
|  |  |  |  |
| Don’t know symptoms | | | |
| Non-RBA  RBA | 76 (84.4) / 14  188 (90.4) / 20 | 1.00  1.73 (0.83, 3.60) | 1.00  1.86 (0.83, 4.19) |
| *P value* |  | *0.14* | *0.13* |
|  |  |  |  |

^1^ For caste, gender, education, food sufficiency, marital status, birth in last 3 years, number of children in household; ^2^ Outcome is “positive” i.e. negative response to “Don’t know symptoms”

**Table 4d: Odds ratio (95% confidence interval) for knowledge of STD treatment amongst 298 participants who had heard of STD**

|  | N (%) yes / no | Unadjusted | Multiply adjusted^1^ |
| --- | --- | --- | --- |
|  |  |  |  |
| Health institution | | | |
| Non-RBA  RBA | 84 (93.3) / 6  202 (97.1) / 6 | 1.00  2.40 (0.75, 7.67) | 1.00  2.67 (0.71, 10.07) |
| *P value* |  | *0.14* | *0.15* |
|  |  |  |  |
| Drug retailers | | | |
| Non-RBA  RBA | 35 (38.9) / 55  82 (39.4) / 126 | 1.00  1.02 (0.62, 1.70) | 1.00  1.01 (0.57, 1.77) |
| *P value* |  | *0.93* | *0.97* |
|  |  |  |  |
| Dhami jhankri | | | |
| Non-RBA  RBA | 3 (3.3) / 87  7 (3.4) / 201 | 1.00  1.01 (0.26, 4.00) | 1.00  1.36 (0.29, 6.25) |
| *P value* |  | *0.99* | *0.70* |
|  |  |  |  |
| Private clinic | | | |
| Non-RBA  RBA | 30 (33.3) / 60  52 (25.0) / 156 | 1.00  0.67 (0.39, 1.14) | 1.00  0.46 (0.25, 0.86) |
| *P value* |  | *0.14* | *0.02* |
|  |  |  |  |
| Home herbal | | | |
| Non-RBA  RBA | 1 (1.1) / 89  8 (3.9) / 200 | 1.00  3.56 (0.44, 28.9) | 1.00  2.29 (0.24, 22.11) |
| *P value* |  | *0.24* | *0.48* |
|  |  |  |  |
|  | N (%) no / yes^2^ | Unadjusted | Multiply adjusted^1^ |
|  |  |  |  |
| Don’t know about treatment | | | |
| Non-RBA  RBA | 84 (93.3) / 6  202 (97.1) / 6 | 1.00  2.40 (0.75, 7.67) | 1.00  2.67 (0.71, 10.07) |
| *P value* |  | *0.14* | *0.15* |
|  |  |  |  |

^1^ For caste, gender, education, food sufficiency, marital status, birth in last 3 years, number of children in household; ^2^ Outcome is “positive” i.e. negative response to “Don’t know about treatment”

**Additional File: Diarrhoeal Diseases**

**Table 5a:** Odds ratio (95% confidence interval) for knowledge of diarrhoea treatment amongst all 628 participants

|  | N (%) yes / no | Unadjusted | Multiply adjusted^1^ |
| --- | --- | --- | --- |
|  |  |  |  |
| Give navajeevan | | | |
| Non-RBA  RBA | 230 (74.0) / 81  257 (81.1) / 60 | 1.00  1.51 (1.03, 2.20) | 1.00  1.33 (0.90, 1.98) |
| *P value* |  | *0.03* | *0.16* |
|  |  |  |  |
| Give home made soup | | | |
| Non-RBA  RBA | 52 (16.7) / 259  78 (24.6) / 239 | 1.00  1.63 (1.10, 2.41) | 1.00  1.64 (1.08, 2.49) |
| *P value* |  | *0.02* | *0.02* |
|  |  |  |  |
| Noon, chini pani | | | |
| Non-RBA  RBA | 29 ( 9.3) / 282  47 (14.8) / 270 | 1.00  1.69 (1.03, 2.77) | 1.00  1.74 (1.04, 2.89) |
| *P value* |  | *0.04* | *0.03* |
|  |  |  |  |
| Take to health institution | | | |
| Non-RBA  RBA | 127 (40.8) / 184  135 (42.6) / 182 | 1.00  1.07 (0.78, 1.48) | 1.00  1.03 (0.74, 1.44) |
| *P value* |  | *0.66* | *0.85* |
|  |  |  |  |
| Provide medicine | | | |
| Non-RBA  RBA | 32 (10.3) / 279  54 (17.0) / 263 | 1.00  1.79 (1.12, 2.86) | 1.00  1.72 (1.05, 2.81) |
| *P value* |  | *0.02* | *0.03* |
|  |  |  |  |
| Injection | | | |
| Non-RBA  RBA | 2 (0.6) / 309  3 (1.0) / 314 | 1.00  1.48 (0.24, 8.89) | 1.00  2.04 (0.26, 16.23) |
| *P value* |  | *0.67* | *0.50* |
|  |  |  |  |
| Dhami jhankri | | | |
| Non-RBA  RBA | 10 (3.2) / 301  9 (2.8) / 308 | 1.00  0.88 (0.35, 2.19) | 1.00  0.76 (0.29, 1.99) |
| *P value* |  | *0.78* | *0.58* |
|  |  |  |  |
| Household remedy | | | |
| Non-RBA  RBA | 40 (12.9) / 271  38 (12.0) / 279 | 1.00  0.92 (0.57, 1.48) | 1.00  0.80 (0.49, 1.33) |
| *P value* |  | *0.74* | *0.39* |
|  |  |  |  |
|  | N (%) no / yes^2^ | Unadjusted | Multiply adjusted^1^ |
|  |  |  |  |
| Don’t know how to treat | | | |
| Non-RBA  RBA | 311 (100.0) / 0  315 ( 99.4) / 2 | - | - |
| *P value* |  |  |  |
|  |  |  |  |

^1^ For caste, gender, education, food sufficiency, marital status, birth in last 3 years, number of children in household; ^2^ Outcome is “positive” i.e. negative response to “Don’t know how to treat”

**Table 5b:** **Odds ratio (95% confidence interval) for knowledge of reasons for feeding Jeevan Jal (oral rehydration solution) amongst all 628 participants**

|  | N (%) yes / no | Unadjusted | Multiply adjusted^1^ |
| --- | --- | --- | --- |
|  |  |  |  |
| To stop diarrhoea | | | |
| Non-RBA  RBA | 250 (80.4) / 61  266 (83.9) / 51 | 1.00  1.27 (0.84, 1.92) | 1.00  1.21 (0.79, 1.85) |
| *P value* |  | *0.25* | *0.38* |
|  |  |  |  |
| To compensate water loss | | | |
| Non-RBA  RBA | 183 (58.8) / 128  239 (75.4) / 78 | 1.00  2.14 (1.52, 3.01) | 1.00  1.96 (1.37, 2.80) |
| *P value* |  | *<0.001* | *<0.001* |
|  |  |  |  |
| To quench thirst | | | |
| Non-RBA  RBA | 56 (18.0) / 255  84 (26.5) / 233 | 1.00  1.64 (1.12, 2.41) | 1.00  1.61 (1.09, 2.40) |
| *P value* |  | *0.01* | *0.02* |
|  |  |  |  |
| To compensate minerals loss | | | |
| Non-RBA  RBA | 9 ( 2.9) / 302  45 (14.2) / 272 | 1.00  5.55 (2.66, 11.57) | 1.00  5.30 (2.50, 11.25) |
| *P value* |  | *<0.001* | *<0.001* |
|  |  |  |  |
|  | N (%) no / yes^2^ | Unadjusted | Multiply adjusted^1^ |
|  |  |  |  |
| Don’t know reasons | | | |
| Non-RBA  RBA | 303 (97.4) / 8  314 (99.1) / 3 | 1.00  2.76 (0.73, 10.51) | 1.00  2.62 (0.65, 10.52) |
| *P value* |  | *0.14* | *0.18* |
|  |  |  |  |

^1^ For caste, gender, education, food sufficiency, marital status, birth in last 3 years, number of children in household; ^2^ Outcome is “positive” i.e. negative response to “Don’t know reasons”

**Table 5c:** **Odds ratio (95% confidence interval) for knowledge of Jeevan Jal (oral rehydration solution) sources amongst all 628 participants**

|  | N (%) yes / no | Unadjusted | Multiply adjusted^1^ |
| --- | --- | --- | --- |
|  |  |  |  |
| Drug retailers | | | |
| Non-RBA  RBA | 183 (58.8) / 128  211 (66.6) / 106 | 1.00  1.39 (1.01, 1.93) | 1.00  1.24 (0.89, 1.75) |
| *P value* |  | *0.05* | *0.21* |
|  |  |  |  |
| Health institution | | | |
| Non-RBA  RBA | 259 (83.3) / 52  288 (90.9) / 29 | 1.00  1.99 (1.23, 3.24) | 1.00  1.94 (1.17, 3.21) |
| *P value* |  | *0.01* | *0.01* |
|  |  |  |  |
| FCHV | | | |
| Non-RBA  RBA | 108 (34.7) / 203  217 (68.5) / 100 | 1.00  4.08 (2.92, 5.69) | 1.00  4.40 (3.09, 6.25) |
| *P value* |  | *<0.001* | *<0.001* |
|  |  |  |  |
| Local shops | | | |
| Non-RBA  RBA | 24 ( 7.7) / 287  42 (13.3) / 275 | 1.00  1.83 (1.08, 3.10) | 1.00  1.72 (0.99, 2.97) |
| *P value* |  | *0.03* | *0.05* |
|  |  |  |  |
|  | N (%) no / yes^2^ | Unadjusted | Multiply adjusted^1^ |
|  |  |  |  |
| Don’t know sources | | | |
| Non-RBA  RBA | 306 ( 98.4) / 5  317 (100.0) / 0 | - | - |
| *P value* |  |  |  |
|  |  |  |  |

^1^ For caste, gender, education, food sufficiency, marital status, birth in last 3 years, number of children in household; ^2^ Outcome is “positive” i.e. negative response to “Don’t know sources”

**Table 5d:** Odds ratio (95% confidence interval) for knowledge of Jeevan Jal (oral rehydration solution) timing and preparation amongst all 628 participants

|  | N (%) yes / no | Unadjusted | Multiply adjusted^1^ |
| --- | --- | --- | --- |
|  |  |  |  |
| Use within 24 hours of preparation (yes vs. no) | | | |
| Non-RBA  RBA | 148 (48.4) / 158  218 (69.0) / 98 | 1.00  2.37 (1.71, 3.29) | 1.00  2.51 (1.77, 3.56) |
| *P value* |  | *<0.001* | *<0.001* |
|  |  |  |  |
| Know how to prepare (yes vs. no) | | | |
| Non-RBA  RBA | 280 (91.5) / 26  283 (89.9) / 32 | 1.00  0.82 (0.48, 1.41) | 1.00  0.89 (0.50, 1.57) |
| *P value* |  | *0.48* | *0.69* |
|  |  |  |  |

^1^ For caste, gender, education, food sufficiency, marital status, birth in last 3 years, number of children in household

**Additional File: Pneumonia**

**Table 6a:** Odds ratio (95% confidence interval) for knowledge of pneumonia symptoms amongst 598 participants who had heard of pneumonia

|  | N (%) yes / no | Unadjusted | Multiply adjusted^1^ |
| --- | --- | --- | --- |
|  |  |  |  |
| Difficulty breathing | | | |
| Non-RBA  RBA | 97 (34.0) / 188  175 (55.9) / 138 | 1.00  2.46 (1.76, 3.42) | 1.00  2.48 (1.75, 3.51) |
| *P value* |  | *<0.001* | *<0.001* |
|  |  |  |  |
| Chest noise | | | |
| Non-RBA  RBA | 190 (66.7) / 95  245 (78.3) / 68 | 1.00  1.80 (1.25, 2.59) | 1.00  1.81 (1.23, 2.65) |
| *P value* |  | *0.002* | *0.002* |
|  |  |  |  |
| Rapid breathing | | | |
| Non-RBA  RBA | 106 (37.2) / 179  158 (50.5) / 155 | 1.00  1.72 (1.24, 2.39) | 1.00  1.71 (1.21, 2.40) |
| *P value* |  | *0.001* | *0.002* |
|  |  |  |  |
| Chest indrowning | | | |
| Non-RBA  RBA | 51 (17.9) / 234  129 (41.2)/ 184 | 1.00  3.22 (2.21, 4.69) | 1.00  2.92 (1.97, 4.33) |
| *P value* |  | *<0.001* | *<0.001* |
|  |  |  |  |
| Fever | | | |
| Non-RBA  RBA | 183 (64.2) / 102  205 (65.5) / 108 | 1.00  1.06 (0.76, 1.48) | 1.00  1.06 (0.75, 1.51) |
| *P value* |  | *0.74* | *0.73* |
|  |  |  |  |
|  | N (%) no / yes^2^ | Unadjusted | Multiply adjusted^1^ |
|  |  |  |  |
| Don’t know symptoms | | | |
| Non-RBA  RBA | 275 (96.5) / 10  307 (98.1) / 6 | 1.00  1.86 (0.67, 5.19) | 1.00  1.53 (0.52, 4.46) |
| *P value* |  | *0.24* | *0.44* |
|  |  |  |  |

^1^ For caste, gender, education, food sufficiency, marital status, birth in last 3 years, number of children in household; ^2^ Outcome is “positive” i.e. negative response to “Don’t know symptoms”

**Table 6b:** Odds ratio (95% confidence interval) for knowledge of pneumonia treatment amongst 598 participants who had heard of pneumonia

|  | N (%) yes / no | Unadjusted | Multiply adjusted^1^ |
| --- | --- | --- | --- |
|  |  |  |  |
| Take to health institution | | | |
| Non-RBA  RBA | 277 (97.2) / 8  307 (98.1) / 6 | 1.00  1.48 (0.51, 4.31) | 1.00  1.24 (0.41, 3.71) |
| *P value* |  | *0.48* | *0.71* |
|  |  |  |  |
| Take to dj | | | |
| Non-RBA  RBA | 17 ( 6.0) / 268  44 (14.1) / 269 | 1.00  2.58 (1.44, 4.63) | 1.00  2.39 (1.30, 4.40) |
| *P value* |  | *0.001* | *0.01* |
|  |  |  |  |
| Home “ailment” (remedy?) | | | |
| Non-RBA  RBA | 54 (19.0) / 231  87 (27.8) / 226 | 1.00  1.65 (1.12, 2.42) | 1.00  1.51 (0.99, 2.29) |
| *P value* |  | *0.01* | *0.05* |
|  |  |  |  |
| Take to FCHV | | | |
| Non-RBA  RBA | 12 ( 4.2) / 273  66 (21.1) / 247 | 1.00  6.08 (3.21, 11.51) | 1.00  7.16 (3.61, 14.20) |
| *P value* |  | *<0.001* | *<0.001* |
|  |  |  |  |
| Drugs retailers | | | |
| Non-RBA  RBA | 75 (26.3) / 210  64 (20.5) / 249 | 1.00  0.72 (0.49, 1.05) | 1.00  0.76 (0.51, 1.14) |
| *P value* |  | *0.09* | *0.19* |
|  |  |  |  |
| Take to private clinic | | | |
| Non-RBA  RBA | 36 (12.6) / 249  62 (19.8) / 251 | 1.00  1.71 (1.09, 2.67) | 1.00  1.49 (0.94, 2.39) |
| *P value* |  | *0.02* | *0.09* |
|  |  |  |  |
|  | N (%) no / yes^2^ | Unadjusted | Multiply adjusted^1^ |
|  |  |  |  |
| Don’t know treatments | | | |
| Non-RBA  RBA | 282 (99.0) / 3  312 (99.7) / 1 | 1.00  3.32 (0.34, 32.09) | 1.00  2.94 (0.28, 30.68) |
| *P value* |  | *0.30* | *0.37* |
|  |  |  |  |

^1^ For caste, gender, education, food sufficiency, marital status, birth in last 3 years, number of children in household; ^2^ Outcome is “positive” i.e. negative response to “Don’t know treatments”

**Table 6c:** **Odds ratio (95% confidence interval) for knowledge of pneumonia medicine sources amongst 598 participants who had heard of pneumonia**

|  | N (%) yes / no | Unadjusted | Multiply adjusted^1^ |
| --- | --- | --- | --- |
|  |  |  |  |
| Health institution | | | |
| Non-RBA  RBA | 274 (96.1) / 11  309 (98.7) / 4 | 1.00  3.10 (0.98, 9.85) | 1.00  3.05 (0.93, 10.00) |
| *P value* |  | *0.06* | *0.07* |
|  |  |  |  |
| Drug retailers | | | |
| Non-RBA  RBA | 153 (53.7) / 132  183 (58.5) / 130 | 1.00  1.21 (0.88, 1.68) | 1.00  1.06 (0.76, 1.49) |
| *P value* |  | *0.24* | *0.73* |
|  |  |  |  |
| FCHV | | | |
| Non-RBA  RBA | 38 (13.3) / 247  107 (34.2) / 206 | 1.00  3.38 (2.23, 5.11) | 1.00  3.63 (2.34, 5.61) |
| *P value* |  | *<0.001* | *<0.001* |
|  |  |  |  |
| MCHW | | | |
| Non-RBA  RBA | 2 (0.7) / 283  21 (6.7) / 292 | 1.00  10.18 (2.36, 43.80) | 1.00  9.29 (2.10, 41.05) |
| *P value* |  | *0.002* | *0.003* |
|  |  |  |  |
| DJ | | | |
| Non-RBA  RBA | 6 (2.1) / 279  7 (2.2) / 306 | 1.00  1.06 (0.35, 3.20) | 1.00  0.91 (0.29, 2.89) |
| *P value* |  | *0.91* | *0.88* |
|  |  |  |  |
|  | N (%) no / yes^2^ | Unadjusted | Multiply adjusted^1^ |
|  |  |  |  |
| Don’t know sources | | | |
| Non-RBA  RBA | 282 (98.9) / 3  312 (99.7) / 1 | 1.00  3.32 (0.34, 32.09) | 1.00  2.39 (0.24, 24.24) |
| *P value* |  | *0.30* | *0.46* |
|  |  |  |  |

^1^ For caste, gender, education, food sufficiency, marital status, birth in last 3 years, number of children in household; ^2^ Outcome is “positive” i.e. negative response to “Don’t know sources”

**Table 6d**: Odds ratio (95% confidence interval) for knowledge of pneumonia prevention amongst 598 participants who had heard of pneumonia

|  | N (%) yes / no | Unadjusted | Multiply adjusted^1^ |
| --- | --- | --- | --- |
|  |  |  |  |
| Keep away from dust and smoke | | | |
| Non-RBA  RBA | 112 (39.3) / 173  196 (62.6) / 117 | 1.00  2.59 (1.86, 3.60) | 1.00  2.83 (1.99, 4.01) |
| *P value* |  | *<0.001* | *<0.001* |
|  |  |  |  |
| Protect from cold | | | |
| Non-RBA  RBA | 198 (69.5) / 87  274 (87.5) / 39 | 1.00  3.09 (2.03, 4.70) | 1.00  2.86 (1.85, 4.40) |
| *P value* |  | *<0.001* | *<0.001* |
|  |  |  |  |
| Hot soup | | | |
| Non-RBA  RBA | 91 (31.9) / 194  141 (45.1) / 172 | 1.00  1.75 (1.25, 2.44) | 1.00  1.75 (1.23, 2.49) |
| *P value* |  | *0.001* | *0.002* |
|  |  |  |  |
| Provide mother’s milk | | | |
| Non-RBA  RBA | 31 (10.9) / 254  114 (36.4) / 199 | 1.00  4.69 (3.03, 7.38) | 1.00  4.50 (2.86, 7.07) |
| *P value* |  | *<0.001* | *<0.001* |
|  |  |  |  |
| Vaccination | | | |
| Non-RBA  RBA | 2 (0.7) / 283  29 (9.3) / 284 | 1.00  14.45 (3.42, 61.13) | 1.00  12.69 (2.93, 54.95) |
| *P value* |  | *<0.001* | *0.001* |
|  |  |  |  |
|  | N (%) no / yes^2^ | Unadjusted | Multiply adjusted^1^ |
|  |  |  |  |
| Don’t know how to prevent | | | |
| Non-RBA  RBA | 250 (87.7) / 35  308 (98.4) / 5 | 1.00  8.62 (3.33, 22.34) | 1.00  8.80 (3.33, 23.2) |
| *P value* |  | *<0.001* | *<0.001* |
|  |  |  |  |

^1^ For caste, gender, education, food sufficiency, marital status, birth in last 3 years, number of children in household; ^2^ Outcome is “positive” i.e. negative response to “Don’t know how to prevent”

**Table 7a:** Odds ratio (95% confidence interval) for knowledge of malaria symptoms amongst 477 participants who had heard of malaria

|  | N (%) yes / no | Unadjusted | Multiply adjusted^1^ |
| --- | --- | --- | --- |
|  |  |  |  |
| Cold with fever | | | |
| Non-RBA  RBA | 131 (59.3) / 90  183 (71.5) / 73 | 1.00  1.72 (1.18, 2.52) | 1.00  1.57 (1.05, 2.34) |
| *P value* |  | *0.005* | *0.03* |
|  |  |  |  |
| Severe fever | | | |
| Non-RBA  RBA | 162 (73.3) / 59  198 (77.3) / 58 | 1.00  1.24 (0.82, 1.89) | 1.00  1.12 (0.72, 1.74) |
| *P value* |  | *0.31* | *0.62* |
|  |  |  |  |
| Severe sweating | | | |
| Non-RBA  RBA | 33 (14.9) / 188  70 (27.3) / 186 | 1.00  2.14 (1.35, 3.40) | 1.00  2.16 (1.33, 3.49) |
| *P value* |  | *0.001* | *0.002* |
|  |  |  |  |
|  | N (%) no / yes^2^ | Unadjusted | Multiply adjusted^1^ |
|  |  |  |  |
| Don’t know symptoms | | | |
| Non-RBA  RBA | 202 (91.4) / 19  245 (95.7) / 11 | 1.00  2.09 (0.97, 4.50) | 1.00  1.96 (0.87, 4.39) |
| *P value* |  | *0.06* | *0.11* |
|  |  |  |  |

^1^ For caste, gender, education, food sufficiency, marital status, birth in last 3 years, number of children in household; ^2^ Outcome is “positive” i.e. negative response to “Don’t know symptoms”

**Table 7b:** Odds ratio (95% confidence interval) for knowledge of malaria transmission amongst 477 participants who had heard of malaria

|  | N (%) yes / no | Unadjusted | Multiply adjusted^1^ |
| --- | --- | --- | --- |
|  |  |  |  |
| Mosquito bite | | | |
| Non-RBA  RBA | 179 (81.0) / 42  214 (83.6) / 42 | 1.00  1.20 (0.75, 1.92) | 1.00  1.19 (0.72, 1.96) |
| *P value* |  | *0.46* | *0.50* |
|  |  |  |  |
| Small flies (bhusuna) | | | |
| Non-RBA  RBA | 25 (11.3) / 196  30 (11.7) / 226 | 1.00  1.04 (0.59, 1.83) | 1.00  1.28 (0.69, 2.38) |
| *P value* |  | *0.89* | *0.43* |
|  |  |  |  |
| Flies | | | |
| Non-RBA  RBA | 12 ( 5.4) / 209  29 (11.3) / 227 | 1.00  2.23 (1.11, 4.47) | 1.00  2.18 (1.06, 4.52) |
| *P value* |  | *0.03* | *0.04* |
|  |  |  |  |
| Contaminated blood | | | |
| Non-RBA  RBA | 6 (2.7) / 215  15 (5.9) / 241 | 1.00  2.23 (0.85, 5.85) | 1.00  2.35 (0.85, 6.45) |
| *P value* |  | *0.10* | *0.10* |
|  |  |  |  |
| Waste | | | |
| Non-RBA  RBA | 37 (16.7) / 184  56 (21.9) / 200 | 1.00  1.39 (0.88, 2.21) | 1.00  1.29 (0.79, 2.09) |
| *P value* |  | *0.16* | *0.31* |
|  |  |  |  |
|  | N (%) no / yes^2^ | Unadjusted | Multiply adjusted^1^ |
|  |  |  |  |
| Don’t know how transmitted | | | |
| Non-RBA  RBA | 182 (82.4) / 39  220 (85.9) / 36 | 1.00  1.31 (0.80, 2.15) | 1.00  1.37 (0.81, 2.31) |
| *P value* |  | *0.28* | *0.24* |
|  |  |  |  |

^1^ For caste, gender, education, food sufficiency, marital status, birth in last 3 years, number of children in household; ^2^ Outcome is “positive” i.e. negative response to “Don’t know how transmitted”

**Table 7c:** Odds ratio (95% confidence interval) for knowledge of malaria medicine source amongst 477 participants who had heard of malaria

|  | N (%) yes / no | Unadjusted | Multiply adjusted^1^ |
| --- | --- | --- | --- |
|  |  |  |  |
| Health institution | | | |
| Non-RBA  RBA | 209 (94.6) / 12  249 (97.3) / 7 | 1.00  2.04 (0.79, 5.28) | 1.00  1.69 (0.62, 4.57) |
| *P value* |  | *0.14* | *0.30* |
|  |  |  |  |
| Drug retailers | | | |
| Non-RBA  RBA | 122 (55.2) / 99  128 (50.0) / 128 | 1.00  0.81 (0.57, 1.16) | 1.00  0.69 (0.47, 1.02) |
| *P value* |  | *0.26* | *0.06* |
|  |  |  |  |
| Dhami jhankri | | | |
| Non-RBA  RBA | 0 (0.0) / 221  7 (2.7) / 249 | - | - |
| *P value* |  |  |  |
|  |  |  |  |
| Private clinic | | | |
| Non-RBA  RBA | 17 ( 7.7) / 204  45 (17.6) / 211 | 1.00  2.56 (1.42, 4.62) | 1.00  2.42 (1.29, 4.54) |
| *P value* |  | *0.002* | *0.01* |
|  |  |  |  |
|  | N (%) no / yes^2^ | Unadjusted | Multiply adjusted^1^ |
|  |  |  |  |
| Don’t know source | | | |
| Non-RBA  RBA | 213 (96.4) / 8  253 (98.8) / 3 | 1.00  3.17 (0.83, 12.09) | 1.00  2.92 (0.73, 11.76) |
| *P value* |  | *0.09* | *0.13* |
|  |  |  |  |

^1^ For caste, gender, education, food sufficiency, marital status, birth in last 3 years, number of children in household; ^2^ Outcome is “positive” i.e. negative response to “Don’t know source”

**Table 7d:** Odds ratio (95% confidence interval) for knowledge of malaria prevention amongst 477 participants who had heard of malaria

|  | N (%) yes / no | Unadjusted | Multiply adjusted^1^ |
| --- | --- | --- | --- |
|  |  |  |  |
| Use net while sleeping | | | |
| Non-RBA  RBA | 194 (87.8) / 27  243 (94.9) / 13 | 1.00  2.60 (1.31, 5.18) | 1.00  2.24 (1.09, 4.60) |
| *P value* |  | *0.01* | *0.03* |
|  |  |  |  |
| Cover body with cloth | | | |
| Non-RBA  RBA | 42 (19.0) / 179  98 (38.3) / 158 | 1.00  2.64 (1.74, 4.02) | 1.00  2.79 (1.79, 4.34) |
| *P value* |  | *<0.001* | *<0.001* |
|  |  |  |  |
| Use oil on the body | | | |
| Non-RBA  RBA | 55 (24.9) / 166  76 (29.7) / 180 | 1.00  1.27 (0.85, 1.91) | 1.00  1.29 (0.84, 1.99) |
| *P value* |  | *0.24* | *0.24* |
|  |  |  |  |
| Use insecticide | | | |
| Non-RBA  RBA | 4 ( 1.8) / 217  41 (16.0) / 215 | 1.00  10.35 (3.64, 29.38) | 1.00  10.71 (3.69, 31.11) |
| *P value* |  | *<0.001* | *<0.001* |
|  |  |  |  |
| Use mosquito repellent | | | |
| Non-RBA  RBA | 40 (18.1) / 181  76 (29.7) / 180 | 1.00  1.91 (1.24, 2.95) | 1.00  1.68 (1.07, 2.65) |
| *P value* |  | *0.004* | *0.03* |
|  |  |  |  |
|  | N (%) no / yes^2^ | Unadjusted | Multiply adjusted^1^ |
|  |  |  |  |
| Don’t know how to prevent | | | |
| Non-RBA  RBA | 201 (91.0) / 20  246 (96.1) / 10 | 1.00  2.45 (1.12, 5.35) | 1.00  2.09 (0.92, 4.74) |
| *P value* |  | *0.03* | *0.08* |
|  |  |  |  |

^1^ For caste, gender, education, food sufficiency, marital status, birth in last 3 years, number of children in household; ^2^ Outcome is “positive” i.e. negative response to “Don’t know how to prevent”

**Additional File: Toilets, Waste & Water**

**Table 8a:** Odds ratio (95% confidence interval) for toilet location amongst all 628 participants

|  | N (%) yes / no | Unadjusted | Multiply adjusted^1^ |
| --- | --- | --- | --- |
|  |  |  |  |
| Surrounding of house | | | |
| Non-RBA  RBA | 33 (10.6) / 278  33 (10.4) / 284 | 1.00  0.98 (0.59, 1.63) | 1.00  0.95 (0.55, 1.64) |
| *P value* |  | *0.94* | *0.85* |
|  |  |  |  |
| River/stream | | | |
| Non-RBA  RBA | 52 (16.7) / 259  52 (16.4) / 265 | 1.00  0.98 (0.64, 1.49) | 1.00  0.69 (0.43, 1.09) |
| *P value* |  | *0.92* | *0.11* |
|  |  |  |  |
| Khet bari | | | |
| Non-RBA  RBA | 79 (25.4) / 232  88 (27.8) / 229 | 1.00  1.13 (0.79, 1.61) | 1.00  1.00 (0.66, 1.51) |
| *P value* |  | *0.50* | *1.00* |
|  |  |  |  |
| Jungle | | | |
| Non-RBA  RBA | 19 (6.1) / 292  7 (2.2) / 310 | 1.00  0.35 (0.14, 0.84) | 1.00  0.28 (0.11, 0.69) |
| *P value* |  | *0.02* | *0.01* |
|  |  |  |  |
| Other person’s toilet | | | |
| Non-RBA  RBA | 4 (1.3) / 307  1 (0.3) / 316 | 1.00  0.24 (0.03, 2.19) | 1.00  0.51 (0.04, 5.98) |
| *P value* |  | *0.21* | *0.60* |
|  |  |  |  |
| Public toilet | | | |
| Non-RBA  RBA | 0 (0.0) / 311  2 (0.6) / 315 | - | - |
| *P value* |  |  |  |
|  |  |  |  |

^1^ For caste, gender, education, food sufficiency, marital status, birth in last 3 years, number of children in household

**Table 8b:** Odds ratio (95% confidence interval) for waste disposal amongst all 628 participants

|  | N (%) yes / no | Unadjusted | Multiply adjusted^1^ |
| --- | --- | --- | --- |
|  |  |  |  |
| Waste collection pit | | | |
| Non-RBA  RBA | 120 (38.6) / 191  161 (50.8) / 156 | 1.00  1.64 (1.20, 2.26) | 1.00  1.65 (1.17, 2.32) |
| *P value* |  | *0.002* | *0.004* |
|  |  |  |  |
| Kitchen garden and burn | | | |
| Non-RBA  RBA | 35 (11.3) / 276  114 (26.0) / 203 | 1.00  4.43 (2.91, 6.74) | 1.00  3.87 (2.51, 5.97) |
| *P value* |  | *<0.001* | *<0.001* |
|  |  |  |  |
| Spread in bari | | | |
| Non-RBA  RBA | 205 (65.9) / 106  152 (48.0) / 165 | 1.00  0.48 (0.35, 0.66) | 1.00  0.44 (0.31, 0.62) |
| *P value* |  | *<0.001* | *<0.001* |
|  |  |  |  |
| Waste collection from home | | | |
| Non-RBA  RBA | 0 (0.0) / 311  0 (0.0) / 317 | - | - |
| *P value* |  |  |  |
|  |  |  |  |
| Public waste container | | | |
| Non-RBA  RBA | 0 (0.0) / 311  0 (0.0) / 317 | - | - |
| *P value* |  |  |  |
|  |  |  |  |

^1^ For caste, gender, education, food sufficiency, marital status, birth in last 3 years, number of children in household

**Table 8c:** Odds ratio (95% confidence interval) for water source amongst all 628 participants

|  | N (%) yes / no | Unadjusted | Multiply adjusted^1^ |
| --- | --- | --- | --- |
|  |  |  |  |
| Well | | | |
| Non-RBA  RBA | 22 (7.1) / 289  2 (0.6) / 315 | 1.00  0.08 (0.02, 0.36) | 1.00  0.12 (0.03, 0.52) |
| *P value* |  | *0.001* | *0.01* |
|  |  |  |  |
| Piped water | | | |
| Non-RBA  RBA | 156 (50.2) / 155  173 (54.6) / 144 | 1.00  1.19 (0.87, 1.63) | 1.00  1.39 (0.97, 1.97) |
| *P value* |  | *0.27* | *0.07* |
|  |  |  |  |
| Spring water | | | |
| Non-RBA  RBA | 19 (6.1) / 292  6 (1.9) / 311 | 1.00  0.30 (0.12, 0.75) | 1.00  0.35 (0.13, 0.95) |
| *P value* |  | *0.01* | *0.04* |
|  |  |  |  |
| Stream or river | | | |
| Non-RBA  RBA | 1 (0.3) / 310  22 (6.9) / 295 | 1.00  23.11 (3.10, 172.6) | 1.00  17.36 (2.28, 132.1) |
| *P value* |  | *0.002* | *0.01* |
|  |  |  |  |
| Hand pump | | | |
| Non-RBA  RBA | 116 (37.3) / 195  120 (37.9) / 197 | 1.00  1.02 (0.74, 1.41) | 1.00  0.86 (0.59, 1.25) |
| *P value* |  | *0.89* | *0.42* |
|  |  |  |  |
| Shallow well | | | |
| Non-RBA  RBA | 44 (14.2) / 267  74 (23.3) / 243 | 1.00  1.85 (1.22, 2.79) | 1.00  1.62 (1.04, 2.52) |
| *P value* |  | *0.003* | *0.03* |
|  |  |  |  |

^1^ For caste, gender, education, food sufficiency, marital status, birth in last 3 years, number of children in household

**Additional File: Reproductive Health**

**Table 9a:** Odds ratio (95% confidence interval) for knowledge of antenatal visit purpose amongst all 628 participants

|  | N (%) yes / no | Unadjusted | Multiply adjusted^1^ |
| --- | --- | --- | --- |
|  |  |  |  |
| Blood pressure check up | | | |
| Non-RBA  RBA | 110 (35.4) / 201  188 (59.3) / 129 | 1.00  2.66 (1.93, 3.68) | 1.00  2.30 (1.64, 3.22) |
| *P value* |  | *<0.001* | *<0.001* |
|  |  |  |  |
| Check child’s status | | | |
| Non-RBA  RBA | 179 (57.6) / 132  235 (74.1) / 82 | 1.00  2.11 (1.51, 2.96) | 1.00  2.11 (1.48, 3.00) |
| *P value* |  | *<0.001* | *<0.001* |
|  |  |  |  |
| Provide iron tablets | | | |
| Non-RBA  RBA | 127 (40.8) / 184  192 (60.6) / 125 | 1.00  2.23 (1.62, 3.06) | 1.00  2.12 (1.51, 2.99) |
| *P value* |  | *<0.001* | *<0.001* |
|  |  |  |  |
| Provide TT injection | | | |
| Non-RBA  RBA | 97 (31.2) / 214  184 (58.0) / 133 | 1.00  3.05 (2.20, 4.24) | 1.00  3.26 (2.29, 4.64) |
| *P value* |  | *<0.001* | *<0.001* |
|  |  |  |  |
| Deworming | | | |
| Non-RBA  RBA | 30 ( 9.7) / 281  61 (19.2) / 256 | 1.00  2.23 (1.40, 3.57) | 1.00  2.26 (1.37, 3.75) |
| *P value* |  | *0.001* | *0.002* |
|  |  |  |  |
|  | N (%) no / yes^2^ | Unadjusted | Multiply adjusted^1^ |
|  |  |  |  |
| Don’t know reasons | | | |
| Non-RBA  RBA | 225 (72.4) / 86  287 (90.5) / 30 | 1.00  3.66 (2.33, 5.74) | 1.00  3.48 (2.16, 5.59) |
| *P value* |  | *<0.001* | *<0.001* |
|  |  |  |  |

^1^ For caste, gender, education, food sufficiency, marital status, birth in last 3 years, number of children in household; ^2^ Outcome is “positive” i.e. negative response to “Don’t know reasons”

**Table 9b:** Odds ratio (95% confidence interval) for knowledge of precautions in pregnancy amongst all 628 participants

|  | N (%) yes / no | Unadjusted | Multiply adjusted^1^ |
| --- | --- | --- | --- |
|  |  |  |  |
| Take adequate rest | | | |
| Non-RBA  RBA | 191 (61.4) / 120  244 (77.0) / 73 | 1.00  2.10 (1.48, 2.97) | 1.00  2.20 (1.53, 3.16) |
| *P value* |  | *<0.001* | *<0.001* |
|  |  |  |  |
| No heavy lifting | | | |
| Non-RBA  RBA | 241 (77.5) / 70  294 (92.7) / 23 | 1.00  3.71 (2.25, 6.13) | 1.00  3.48 (2.08, 5.83) |
| *P value* |  | *<0.001* | *<0.001* |
|  |  |  |  |
| Take iron tablets | | | |
| Non-RBA  RBA | 92 (29.6) / 219  162 (51.1) / 155 | 1.00  2.49 (1.79, 3.45) | 1.00  2.24 (1.59, 3.16) |
| *P value* |  | *<0.001* | *<0.001* |
|  |  |  |  |
| Share work with family | | | |
| Non-RBA  RBA | 5 (1.6) / 306  29 (9.2) / 288 | 1.00  6.16 (2.35, 16.14) | 1.00  6.37 (2.38, 17.08) |
| *P value* |  | *<0.001* | *<0.001* |
|  |  |  |  |
| No smoking and drinking | | | |
| Non-RBA  RBA | 38 (12.2) / 273  113 (35.7) / 204 | 1.00  3.98 (2.64, 6.00) | 1.00  3.73 (2.44, 5.72) |
| *P value* |  | *<0.001* | *<0.001* |
|  |  |  |  |
|  | N (%) no / yes^2^ | Unadjusted | Multiply adjusted^1^ |
|  |  |  |  |
| Don’t know about precautions | | | |
| Non-RBA  RBA | 269 (86.5) / 42  305 (95.2) / 12 | 1.00  3.97 (2.05, 7.69) | 1.00  3.80 (1.91, 7.57) |
| *P value* |  | *<0.001* | *<0.001* |
|  |  |  |  |

^1^ For caste, gender, education, food sufficiency, marital status, birth in last 3 years, number of children in household; ^2^ Outcome is “positive” i.e. negative response to “Don’t know about precautions”

**Table 9c:** Odds ratio (95% confidence interval) for knowledge of specific pregnancy danger signs amongst 510 participants who were aware of pregnancy danger signs

|  | N (%) yes / no | Unadjusted | Multiply adjusted^1^ |
| --- | --- | --- | --- |
|  |  |  |  |
| Excessive bleeding | | | |
| Non-RBA  RBA | 181 (83.4) / 36  257 (87.7) / 36 | 1.00  1.42 (0.86, 2.34) | 1.00  1.40 (0.83, 2.36) |
| *P value* |  | *0.17* | *0.21* |
|  |  |  |  |
| Swelling of limbs | | | |
| Non-RBA  RBA | 77 (35.5) / 140  164 (56.0) / 129 | 1.00  2.31 (1.61, 3.32) | 1.00  2.26 (1.55, 3.29) |
| *P value* |  | *<0.001* | *<0.001* |
|  |  |  |  |
| Severe headache | | | |
| Non-RBA  RBA | 87 (40.1) / 130  142 (48.5) / 151 | 1.00  1.41 (0.99, 2.00) | 1.00  1.26 (0.87, 1.83) |
| *P value* |  | *0.06* | *0.22* |
|  |  |  |  |
| Lower abdominal pain | | | |
| Non-RBA  RBA | 88 (40.6) / 129  152 (51.9) / 141 | 1.00  1.58 (1.11, 2.25) | 1.00  1.84 (1.26, 2.68) |
| *P value* |  | *0.01* | *0.002* |
|  |  |  |  |
| Unconsciousness | | | |
| Non-RBA  RBA | 40 (18.4) / 177  107 (36.5) / 186 | 1.00  2.55 (1.68, 3.86) | 1.00  2.57 (1.65, 4.01) |
| *P value* |  | *<0.001* | *<0.001* |
|  |  |  |  |
| Fever | | | |
| Non-RBA  RBA | 41 (18.9) / 176  79 (27.0) / 214 | 1.00  1.58 (1.03, 2.43) | 1.00  1.82 (1.16, 2.86) |
| *P value* |  | *0.03* | *0.01* |
|  |  |  |  |
| Poor vision | | | |
| Non-RBA  RBA | 32 (14.8) / 185  58 (19.8) / 235 | 1.00  1.43 (0.89, 2.29) | 1.00  1.07 (0.65, 1.79) |
| *P value* |  | *0.14* | *0.78* |
|  |  |  |  |
| Foul smelling discharge from vagina | | | |
| Non-RBA  RBA | 20 ( 9.2) / 197  54 (18.4) / 239 | 1.00  2.23 (1.29, 3.84) | 1.00  1.99 (1.13, 3.51) |
| *P value* |  | *0.004* | *0.02* |
|  |  |  |  |
|  |  |  |  |
|  | N (%) no / yes^2^ | Unadjusted | Multiply adjusted^1^ |
|  |  |  |  |
| Don’t know any symptoms | | | |
| Non-RBA  RBA | 216 (99.5) / 1  292 (99.7) / 1 | 1.00  1.35 (0.08, 21.73) | 1.00  1.11 (0.05, 22.73) |
| *P value* |  | *0.83* | *0.95* |
|  |  |  |  |

^1^ For caste, gender, education, food sufficiency, marital status, birth in last 3 years, number of children in household; ^2^ Outcome is “positive” i.e. negative response to “Don’t know any symptoms”

**Table 9d:** Odds ratio (95% confidence interval) for knowledge of birth preparedness amongst all 242 participants who were aware of BPP

|  | N (%) yes / no | Unadjusted | Multiply adjusted^1^ |
| --- | --- | --- | --- |
|  |  |  |  |
| Preparing to take help from SBA | | | |
| Non-RBA  RBA | 28 (40.0) / 42  82 (47.7) / 90 | 1.00  1.37 (0.78, 2.40) | 1.00  1.24 (0.65, 2.36) |
| *P value* |  | *0.28* | *0.51* |
|  |  |  |  |
| Knowing health institution for delivery | | | |
| Non-RBA  RBA | 14 (20.0) / 56  67 (39.0) / 105 | 1.00  2.55 (1.32, 4.94) | 1.00  2.04 (0.98, 4.27) |
| *P value* |  | *0.01* | *0.06* |
|  |  |  |  |
| Arranging money for delivery | | | |
| Non-RBA  RBA | 60 (85.7) / 10  148 (86.1) / 24 | 1.00  1.03 (0.46, 2.28) | 1.00  1.40 (0.54, 3.63) |
| *P value* |  | *0.95* | *0.49* |
|  |  |  |  |
| Arranging for transport | | | |
| Non-RBA  RBA | 15 (21.4) / 55  42 (24.4) / 130 | 1.00  1.18 (0.61, 2.31) | 1.00  1.15 (0.54, 2.46) |
| *P value* |  | *0.62* | *0.71* |
|  |  |  |  |
| 3 persons for blood donation | | | |
| Non-RBA  RBA | 0 ( 0.0) / 70  22 (12.8) / 150 | - | - |
| *P value* |  |  |  |
|  |  |  |  |
|  | N (%) no / yes^2^ | Unadjusted | Multiply adjusted^1^ |
|  |  |  |  |
| Don’t know about specific preparations | | | |
| Non-RBA  RBA | 70 (100.0) / 0  170 ( 98.8) / 2 | - | - |
| *P value* |  |  |  |
|  |  |  |  |

^1^ For caste, gender, education, food sufficiency, marital status, birth in last 3 years, number of children in household; ^2^ Outcome is “positive” i.e. negative response to “Don’t know about specific preparations”

**Table 9e:** Odds ratio (95% confidence interval) for delivery details amongst all 181 participants who delivered a baby in the last 3 years

|  | N (%) | Unadjusted | Multiply adjusted^1^ |
| --- | --- | --- | --- |
|  |  |  |  |
| Place of delivery (Health institution vs. home) | | | |
| Non-RBA  RBA | 15 (19.7) / 61  25 (24.0) / 79 | 1.00  1.29 (0.63, 2.65) | 1.00  1.29 (0.58, 2.84) |
| *P value* |  | *0.49* | *0.53* |
|  |  |  |  |
| Person involved (Skilled birth attendant vs. other) | | | |
| Non-RBA  RBA | 11 (14.5) / 65  15 (14.3) / 90 | 1.00  0.98 (0.42, 2.28) | 1.00  0.88 (0.35, 2.21) |
| *P value* |  | *0.97* | *0.79* |
|  |  |  |  |
| Used clean home deliver kit (Yes vs. no) | | | |
| Non-RBA  RBA | 43 (56.6) / 33  55 (52.4) / 50 | 1.00  0.84 (0.47, 1.53) | 1.00  0.86 (0.45, 1.66) |
| *P value* |  | *0.58* | *0.66* |
|  |  |  |  |

^1^ For caste, gender, education, food sufficiency, marital status, number of children in household

**Table 9f:** Odds ratio (95% confidence interval) for knowledge of specific delivery danger signs amongst 498 participants who were aware of pregnancy danger signs

|  | N (%) yes / no | Unadjusted | Multiply adjusted^1^ |
| --- | --- | --- | --- |
|  |  |  |  |
| Excessive bleeding | | | |
| Non-RBA  RBA | 192 (86.9) / 29  254 (91.7) / 23 | 1.00  1.67 (0.94, 2.97) | 1.00  1.75 (0.95, 3.21) |
| *P value* |  | *0.08* | *0.07* |
|  |  |  |  |
| Unconsciousness | | | |
| Non-RBA  RBA | 32 (14.5) / 189  114 (41.2) / 163 | 1.00  4.13 (2.65, 6.44) | 1.00  4.23 (2.65, 6.75) |
| *P value* |  | *<0.001* | *<0.001* |
|  |  |  |  |
| Prolonged labour | | | |
| Non-RBA  RBA | 71 (32.1) / 150  130 (46.9) / 147 | 1.00  1.87 (1.29, 2.70) | 1.00  2.09 (1.41, 3.10) |
| *P value* |  | *0.001* | *<0.001* |
|  |  |  |  |
| Child’s position | | | |
| Non-RBA  RBA | 72 (32.6) / 149  112 (40.4) / 165 | 1.00  1.40 (0.97, 2.03) | 1.00  1.44 (0.97, 2.12) |
| *P value* |  | *0.07* | *0.07* |
|  |  |  |  |
| Excessive fluid discharge | | | |
| Non-RBA  RBA | 17 ( 7.7) / 204  54 (19.5) / 223 | 1.00  2.91 (1.63, 5.18) | 1.00  2.60 (1.42, 4.75) |
| *P value* |  | *<0.001* | *0.002* |
|  |  |  |  |
| Swelling of limbs | | | |
| Non-RBA  RBA | 51 (23.1) / 170  93 (33.6) / 184 | 1.00  1.68 (1.13, 2.51) | 1.00  1.49 (0.97, 2.28) |
| *P value* |  | *0.01* | *0.07* |
|  |  |  |  |
| Child’s hand or feet coming out first | | | |
| Non-RBA  RBA | 87 (39.4) / 134  124 (44.8) / 153 | 1.00  1.25 (0.87, 1.79) | 1.00  1.32 (0.90, 1.94) |
| *P value* |  | *0.23* | *0.15* |
|  |  |  |  |
| Fever | | | |
| Non-RBA  RBA | 8 (3.6) / 213  25 (9.0) / 252 | 1.00  2.64 (1.17, 5.98) | 1.00  2.16 (0.91, 5.13) |
| *P value* |  | *0.02* | *0.08* |
|  |  |  |  |
| Umbilical cord comes first | | | |
| Non-RBA  RBA | 51 (23.1) / 170  92 (33.2) / 185 | 1.00  1.66 (1.11, 2.47) | 1.00  1.72 (1.11, 2.68) |
| *P value* |  | *0.01* | *0.02* |
|  |  |  |  |
| Delay in placenta delivery | | | |
| Non-RBA  RBA | 1 (0.5) / 220  17 (6.1) / 260 | 1.00  14.38 (1.90, 109.0) | 1.00  15.09 (1.92, 118.9) |
| *P value* |  | *0.01* | *0.01* |
|  |  |  |  |
| Delayed delivery with 12 hours of labour | | | |
| Non-RBA  RBA | 1 (0.5) / 220  7 (2.5) / 270 | 1.00  5.70 (0.70, 46.71) | 1.00  5.69 (0.64, 50.46) |
| *P value* |  | *0.11* | *0.12* |
|  |  |  |  |
|  | N (%) no / yes^2^ | Unadjusted | Multiply adjusted^1^ |
|  |  |  |  |
| Don’t know about specific danger signs | | | |
| Non-RBA  RBA | 218 ( 98.6) / 3  277 (100.0) / 0 | - | - |
| *P value* |  |  |  |
|  |  |  |  |

^1^ For caste, gender, education, food sufficiency, marital status, birth in last 3 years, number of children in household; ^2^ Outcome is “positive” i.e. negative response to “Don’t know about specific danger signs”

**Table 9g:** Odds ratio (95% confidence interval) for knowledge of newborn care amongst all 628 participants

|  | N (%) yes / no | Unadjusted | Multiply adjusted^1^ |
| --- | --- | --- | --- |
|  |  |  |  |
| Clean the baby | | | |
| Non-RBA  RBA | 168 (54.0) / 143  238 (75.1) / 79 | 1.00  2.56 (1.83, 3.60) | 1.00  2.35 (1.66, 3.34) |
| *P value* |  | *<0.001* | *<0.001* |
|  |  |  |  |
| Keep the baby warm | | | |
| Non-RBA  RBA | 103 (33.1) / 208  160 (50.5) / 157 | 1.00  2.06 (1.49, 2.84) | 1.00  1.88 (1.33, 2.66) |
| *P value* |  | *<0.001* | *<0.001* |
|  |  |  |  |
| No bathing for 24 hours | | | |
| Non-RBA  RBA | 22 ( 7.1) / 289  82 (25.9) / 235 | 1.00  4.58 (2.78, 7.57) | 1.00  4.36 (2.60, 7.32) |
| *P value* |  | *<0.001* | *<0.001* |
|  |  |  |  |
| Feed mother’s milk | | | |
| Non-RBA  RBA | 115 (37.0) / 196  179 (56.5) / 138 | 1.00  2.21 (1.61, 3.04) | 1.00  2.37 (1.68, 3.36) |
| *P value* |  | *<0.001* | *<0.001* |
|  |  |  |  |
| Cut umbilical cord and keep dry | | | |
| Non-RBA  RBA | 135 (43.4) / 176  183 (57.7) / 134 | 1.00  1.78 (1.30, 2.44) | 1.00  1.81 (1.30, 2.53) |
| *P value* |  | *<0.001* | *<0.001* |
|  |  |  |  |
| BCG | | | |
| Non-RBA  RBA | 5 (1.6) / 306  23 (7.3) / 294 | 1.00  4.79 (1.80, 12.76) | 1.00  4.84 (1.77, 13.21) |
| *P value* |  | *0.002* | *0.002* |
|  |  |  |  |
|  | N (%) no / yes^2^ | Unadjusted | Multiply adjusted^1^ |
|  |  |  |  |
| Don’t know about specific care | | | |
| Non-RBA  RBA | 284 (91.3) / 27  310 (97.8) / 7 | 1.00  4.21 (1.81, 9.82) | 1.00  3.70 (1.54, 8.88) |
| *P value* |  | *0.001* | *0.003* |
|  |  |  |  |

^1^ For caste, gender, education, food sufficiency, marital status, birth in last 3 years, number of children in household; ^2^ Outcome is “positive” i.e. negative response to “Don’t know about specific care”

**Table 9h:** **Odds ratio (95% confidence interval) for knowledge of newborn danger signs amongst all 628 participants**

|  | N (%) yes / no | Unadjusted | Multiply adjusted^1^ |
| --- | --- | --- | --- |
|  |  |  |  |
| Unable to suck milk | | | |
| Non-RBA  RBA | 166 (54.5) / 145  233 (73.5) / 84 | 1.00  2.42 (1.73, 3.39) | 1.00  2.78 (1.93, 4.00) |
| *P value* |  | *<0.001* | *<0.001* |
|  |  |  |  |
| Lazy or unconscious | | | |
| Non-RBA  RBA | 31 (10.0) / 280  57 (18.0) / 260 | 1.00  1.98 (1.24, 3.16) | 1.00  1.96 (1.20, 3.20) |
| *P value* |  | *0.004* | *0.01* |
|  |  |  |  |
| Low crying or unable to cry | | | |
| Non-RBA  RBA | 42 (13.5) / 269  90 (28.4) / 227 | 1.00  2.54 (1.69, 3.81) | 1.00  2.44 (1.60, 3.71) |
| *P value* |  | *<0.001* | *<0.001* |
|  |  |  |  |
| Rapid breathing | | | |
| Non-RBA  RBA | 45 (14.5) / 266  110 (34.7) / 207 | 1.00  3.14 (2.12, 4.65) | 1.00  3.12 (2.07, 4.70) |
| *P value* |  | *<0.001* | *<0.001* |
|  |  |  |  |
| Pus in naval | | | |
| Non-RBA  RBA | 12 ( 3.9) / 299  72 (22.7) / 245 | 1.00  7.32 (3.88, 13.80) | 1.00  6.67 (3.49, 12.74) |
| *P value* |  | *<0.001* | *<0.001* |
|  |  |  |  |
|  | N (%) no / yes^2^ | Unadjusted | Multiply adjusted^1^ |
|  |  |  |  |
| Don’t know any danger signs | | | |
| Non-RBA  RBA | 189 (60.8) / 122  248 (78.2) / 69 | 1.00  2.32 (1.63, 3.29) | 1.00  2.68 (1.83, 3.92) |
| *P value* |  | *<0.001* | *<0.001* |
|  |  |  |  |

^1^ For caste, gender, education, food sufficiency, marital status, birth in last 3 years, number of children in household; ^2^ Outcome is “positive” i.e. negative response to “Don’t know any danger signs”

**Table 9i:** Odds ratio (95% confidence interval) for knowledge of specific temporary contraceptive methods amongst 602 participants who had heard of family planning

|  | N (%) yes / no | Unadjusted | Multiply adjusted^1^ |
| --- | --- | --- | --- |
|  |  |  |  |
| Condoms | | | |
| Non-RBA  RBA | 195 (67.0) / 96  258 (83.0) / 53 | 1.00  2.40 (1.63, 3.52) | 1.00  3.33 (2.16, 5.14) |
| *P value* |  | *<0.001* | *<0.001* |
|  |  |  |  |
| Sangani | | | |
| Non-RBA  RBA | 247 (84.9) / 44  283 (91.0) / 28 | 1.00  1.80 (1.09, 2.98) | 1.00  1.85 (1.10, 3.14) |
| *P value* |  | *0.02* | *0.02* |
|  |  |  |  |
| Pills | | | |
| Non-RBA  RBA | 245 (84.2) / 46  281 (90.4) / 30 | 1.00  1.76 (1.08, 2.87) | 1.00  2.10 (1.26, 3.50) |
| *P value* |  | *0.02* | *0.004* |
|  |  |  |  |
| Narplant | | | |
| Non-RBA  RBA | 108 (37.1) / 183  174 (56.0) / 137 | 1.00  2.15 (1.55, 2.98) | 1.00  2.43 (1.69, 3.50) |
| *P value* |  | *<0.001* | *<0.001* |
|  |  |  |  |
| Copper T | | | |
| Non-RBA  RBA | 83 (28.5) / 208  156 (50.2) / 155 | 1.00  2.52 (1.80, 3.54) | 1.00  3.03 (2.05, 4.48) |
| *P value* |  | *<0.001* | *<0.001* |
|  |  |  |  |
| Kamal | | | |
| Non-RBA  RBA | 10 ( 3.4) / 281  43 (13.8) / 268 | 1.00  4.51 (2.22, 9.15) | 1.00  4.00 (1.91, 8.36) |
| *P value* |  | *<0.001* | *<0.001* |
|  |  |  |  |
| Sex during safe period | | | |
| Non-RBA  RBA | 1 (0.3) / 290  0 (0.0) / 311 | - | - |
| *P value* |  |  |  |
|  |  |  |  |
| Ejaculating outside | | | |
| Non-RBA  RBA | 0 (0.0) / 291  0 (0.0) / 311 | - | - |
| *P value* |  |  |  |
|  |  |  |  |
|  | N (%) no / yes^2^ | Unadjusted | Multiply adjusted^1^ |
|  |  |  |  |
| Don’t know any temporary methods | | | |
| Non-RBA  RBA | 282 (96.9) / 9  303 (97.4) / 8 | 1.00  1.21 (0.46, 3.18) | 1.00  1.53 (0.57, 4.15) |
| *P value* |  | *0.70* | *0.40* |
|  |  |  |  |

^1^ For caste, gender, education, food sufficiency, marital status, birth in last 3 years, number of children in household; ^2^ Outcome is “positive” i.e. negative response to “Don’t know any temporary methods”

**Table 9j:** Odds ratio (95% confidence interval) for knowledge of specific permanent contraceptive methods amongst 602 participants who had heard of family planning

|  | N (%) yes / no | Unadjusted | Multiply adjusted^1^ |
| --- | --- | --- | --- |
|  |  |  |  |
| Laparoscopy | | | |
| Non-RBA  RBA | 232 (79.7) / 59  272 (87.5) / 39 | 1.00  1.77 (1.14, 2.76) | 1.00  1.81 (1.14, 2.88) |
| *P value* |  | *0.01* | *0.01* |
|  |  |  |  |
| Vasectomy | | | |
| Non-RBA  RBA | 167 (57.4) / 124  238 (76.5) / 73 | 1.00  2.42 (1.71, 3.44) | 1.00  3.39 (2.28, 5.05) |
| *P value* |  | *<0.001* | *<0.001* |
|  |  |  |  |
|  | N (%) no / yes^2^ | Unadjusted | Multiply adjusted^1^ |
|  |  |  |  |
| Don’t know any permanent methods | | | |
| Non-RBA  RBA | 242 (83.2) / 49  274 (88.1) / 37 | 1.00  1.50 (0.95, 2.38) | 1.00  1.53 (0.94, 2.49) |
| *P value* |  | *0.09* | *0.08* |
|  |  |  |  |

^1^ For caste, gender, education, food sufficiency, marital status, birth in last 3 years, number of children in household; ^2^ Outcome is “positive” i.e. negative response to “Don’t know any permanent methods”

**Table 9k:** Odds ratio (95% confidence interval) for knowledge of sources of contraceptive methods amongst 602 participants who had heard of family planning

|  | N (%) yes / no | Unadjusted | Multiply adjusted^1^ | |
| --- | --- | --- | --- | --- |
|  |  |  |  | |
| Health institutions | | | | |
| Non-RBA  RBA | 277 (95.2) / 14  298 (95.8) / 13 | 1.00  1.16 (0.54, 2.51) | 1.00  1.34 (0.60, 3.00) | |
| *P value* |  | *0.71* | *0.47* | |
|  |  |  |  | |
| Outreach clinic | | | | |
| Non-RBA  RBA | 59 (20.8) / 232  132 (42.4) / 179 | 1.00  2.90 (2.02, 4.17) | 1.00  3.08 (2.11, 4.51) | |
| *P value* |  | *<0.001* | *<0.001* | |
|  |  |  |  | |
| Health workers | | | | |
| Non-RBA  RBA | 63 (21.7) / 228  124 (39.9) / 187 | 1.00  2.40 (1.67, 3.44) | 1.00  2.84 (1.92, 4.20) | |
| *P value* |  | *<0.001* | *<0.001* | |
|  |  |  |  | |
| FCHV | | | | |
| Non-RBA  RBA | 72 (24.7) / 219  119 (38.3) / 192 | 1.00  1.89 (1.33, 2.68) | 1.00  1.92 (1.33, 2.77) | |
| *P value* |  | *<0.001* | *<0.001* | |
|  |  |  |  | |
| Drugs retailer | | | | |
| Non-RBA  RBA | 48 (16.5) / 243  97 (31.2) / 214 | 1.00  2.29 (1.55, 3.39) | 1.00  2.35 (1.56, 3.53) | |
| *P value* |  | *<0.001* | *<0.001* | |
|  |  |  |  | |
| Tea shops | | | | |
| Non-RBA  RBA | 1 (0.3) / 290  14 (4.5) / 297 | 1.00  13.67 (1.79, 104.63) | | 1.00  15.48 (1.97, 121.71) |
| *P value* |  | *0.01* | | *0.01* |
|  |  |  | |  |
| Grocery shops | | | | |
| Non-RBA  RBA | 2 (0.7) / 289  9 (2.9) / 302 | 1.00  4.31 (0.92, 20.10) | 1.00  4.35 (0.89, 21.34) | |
| *P value* |  | *0.06* | *0.07* | |
|  |  |  |  | |
| Paan Pasal | | | | |
| Non-RBA  RBA | 0 (0.0) / 291  9 (2.9) / 302 | - | - | |
| *P value* |  |  |  | |
|  |  |  |  | |
|  | N (%) no / yes^2^ | Unadjusted | Multiply adjusted^1^ | |
|  |  |  |  | |
| Don’t know any sources | | | | |
| Non-RBA  RBA | 284 (97.6) / 7  303 (97.4) / 8 | 1.00  0.93 (0.33, 2.61) | 1.00  1.21 (0.42, 3.49) | |
| *P value* |  | *0.90* | *0.73* | |
|  |  |  |  | |

^1^ For caste, gender, education, food sufficiency, marital status, birth in last 3 years, number of children in household; ^2^ Outcome is “positive” i.e. negative response to “Don’t know any sources”

**Table 9l:** Odds ratio (95% confidence interval) for use of specific temporary contraceptive methods amongst 262 participants who use family planning

|  | N (%) yes / no | Unadjusted | Multiply adjusted^1^ | |
| --- | --- | --- | --- | --- |
|  |  |  |  | |
| Condom | | | | |
| Non-RBA  RBA | 4 (3.5) / 109  4 (2.7) / 145 | 1.00  0.75 (0.18, 3.07) | 1.00  0.58 (0.13, 2.66) | |
| *P value* |  | *0.69* | *0.48* | |
|  |  |  |  | |
| Sangani | | | | |
| Non-RBA  RBA | 37 (32.7) / 76  58 (38.9) / 91 | 1.00  1.31 (0.78, 2.19) | 1.00  1.27 (0.73, 2.20) | |
| *P value* |  | *0.30* | *0.40* | |
|  |  |  |  | |
| Pills | | | | |
| Non-RBA  RBA | 17 (15.0) / 96  24 (16.1) / 125 | 1.00  1.08 (0.55, 2.13) | 1.00  0.97 (0.48, 1.97) | |
| *P value* |  | *0.82* | *0.93* | |
|  |  |  |  | |
| Narplant | | | | |
| Non-RBA  RBA | 1 (0.9) / 112  2 (1.3) / 147 | 1.00  1.52 (0.14, 17.0) | 1.00  2.02 (0.16, 26.2) | |
| *P value* |  | *0.73* | *0.59* | |
|  |  |  |  | |
| Copper T | | | | |
| Non-RBA  RBA | 2 (1.8) / 111  0 (0.0) / 149 | - | - | |
| *P value* |  |  |  | |
|  |  |  |  | |
| Kamal | | | | |
| Non-RBA  RBA | 0 (0.0) / 113  2 (1.3) / 147 | - | | - |
| *P value* |  |  | |  |
|  |  |  | |  |
| Sex during safe period | | | | |
| Non-RBA  RBA | 0 (0.0) / 113  0 (0.0) / 149 | - | - | |
| *P value* |  |  |  | |
|  |  |  |  | |
| Ejaculating outside | | | | |
| Non-RBA  RBA | 0 (0.0) / 113  0 (0.0) / 149 | - | - | |
| *P value* |  |  |  | |
|  |  |  |  | |
|  |  |  |  | |

^1^ For caste, gender, education, food sufficiency, marital status, birth in last 3 years, number of children in household

**Table 9m:** Odds ratio (95% confidence interval) for knowledge of specific permanent contraceptive methods amongst 262 participants who use family planning

|  | N (%) yes / no | Unadjusted | Multiply adjusted^1^ |
| --- | --- | --- | --- |
|  |  |  |  |
| Laparoscopy | | | |
| Non-RBA  RBA | 55 (48.7) / 58  57 (38.3) / 92 | 1.00  0.65 (0.40, 1.07) | 1.00  0.66 (0.37, 1.16) |
| *P value* |  | *0.09* | *0.15* |
|  |  |  |  |
| Vasectomy | | | |
| Non-RBA  RBA | 2 (1.8) / 111  6 (4.0) / 143 | 1.00  2.33 (0.46, 11.76) | 1.00  3.59 (0.66, 19.49) |
| *P value* |  | *0.31* | *0.14* |
|  |  |  |  |

^1^ For caste, gender, education, food sufficiency, marital status, birth in last 3 years, number of children in household

**Table 9n:** **Odds ratio (95% confidence interval) for knowledge of legal abortion circumstances amongst all 628 participants**

|  | N (%) yes / no | Unadjusted | Multiply adjusted^1^ |
| --- | --- | --- | --- |
|  |  |  |  |
| Within 12 weeks of pregnancy | | | |
| Non-RBA  RBA | 27 ( 8.7) / 284  56 (17.7) / 261 | 1.00  2.26 (1.38, 3.68) | 1.00  2.60 (1.52, 4.47) |
| *P value* |  | *0.001* | *0.001* |
|  |  |  |  |
| Rape or incest | | | |
| Non-RBA  RBA | 25 ( 8.0) / 286  78 (24.6) / 239 | 1.00  3.73 (2.31, 6.05) | 1.00  3.81 (2.30, 6.33) |
| *P value* |  | *<0.001* | *<0.001* |
|  |  |  |  |
| Threaten mother or child’s life | | | |
| Non-RBA  RBA | 14 ( 4.5) / 297  38 (12.0) / 279 | 1.00  2.89 (1.53, 5.45) | 1.00  3.05 (1.53, 6.09) |
| *P value* |  | *0.001* | *0.002* |
|  |  |  |  |
|  | N (%) no / yes^2^ | Unadjusted | Multiply adjusted^1^ |
|  |  |  |  |
| Don’t know any circumstances | | | |
| Non-RBA  RBA | 65 (20.9) / 246  131 (41.3) / 186 | 1.00  2.67 (1.87, 3.79) | 1.00  3.38 (2.24, 5.09) |
| *P value* |  | *<0.001* | *<0.001* |
|  |  |  |  |

^1^ For caste, gender, education, food sufficiency, marital status, birth in last 3 years, number of children in household; ^2^ Outcome is “positive” i.e. negative response to “Don’t know any circumstances”

**Table 9o:** Odds ratio (95% confidence interval) for knowledge of illegal abortion circumstances amongst all 628 participants

|  | N (%) yes / no | Unadjusted | Multiply adjusted^1^ |
| --- | --- | --- | --- |
|  |  |  |  |
| Without woman’s consent | | | |
| Non-RBA  RBA | 28 ( 9.0) / 283  91 (28.7) / 226 | 1.00  4.07 (2.57, 6.44) | 1.00  4.63 (2.81, 7.62) |
| *P value* |  | *<0.001* | *<0.001* |
|  |  |  |  |
| Sex selection | | | |
| Non-RBA  RBA | 7 ( 2.3) / 304  33 (10.4) / 284 | 1.00  5.05 (2.20, 11.59) | 1.00  4.82 (2.05, 11.32) |
| *P value* |  | *<0.001* | *<0.001* |
|  |  |  |  |
| Conditions prohibited by law | | | |
| Non-RBA  RBA | 7 ( 2.3) / 304  17 ( 5.4) / 300 | 1.00  2.46 (1.01, 6.02) | 1.00  2.25 (0.88, 5.75) |
| *P value* |  | *0.05* | *0.09* |
|  |  |  |  |
|  | N (%) no / yes^2^ | Unadjusted | Multiply adjusted^1^ |
|  |  |  |  |
| Don’t know any circumstances | | | |
| Non-RBA  RBA | 40 (12.9) / 271  113 (35.7) / 204 | 1.00  3.75 (2.51, 5.62) | 1.00  4.32 (2.77, 6.73) |
| *P value* |  | *<0.001* | *<0.001* |
|  |  |  |  |

^1^ For caste, gender, education, food sufficiency, marital status, birth in last 3 years, number of children in household; ^2^ Outcome is “positive” i.e. negative response to “Don’t know any circumstances”

**Table 9p**: Odds ratio (95% confidence interval) for knowledge of TT shots amongst all 628 participants

|  | N (%) yes / no | Unadjusted | Multiply adjusted^1^ |
| --- | --- | --- | --- |
|  |  |  |  |
| 3+ times | | | |
| Non-RBA  RBA | 51 (16.4) / 260  59 (18.6) / 258 | 1.00  1.17 (0.77, 1.76) | 1.00  1.27 (0.82, 1.99) |
| *P value* |  | *0.47* | *0.29* |
|  |  |  |  |

^1^ For caste, gender, education, food sufficiency, marital status, birth in last 3 years, number of children in household

**Additional File:Health Institutions & Health Workers**

**Table10a: Odds ratio (95% confidence interval) for visiting health institute when sick amongst all 628 participants**

|  | N (%) yes / no | Unadjusted | Multiply adjusted^1^ |
| --- | --- | --- | --- |
|  |  |  |  |
| Visit health institute when sick | | | |
| Non-RBA  RBA | 302 (97.1) / 9  313 (98.7) / 4 | 1.00  2.33 (0.71, 7.65) | 1.00  2.47 (0.73, 8.42) |
| *P value* |  | *0.16* | *0.15* |
|  |  |  |  |

^1^ For caste, gender, education, food sufficiency, marital status, birth in last 3 years, number of children in household

**Table 10b**: Odds ratio (95% confidence interval) for knowledge of services available at health institute amongst all 628 participants

|  | N (%) yes / no | Unadjusted | Multiply adjusted^1^ |
| --- | --- | --- | --- |
|  |  |  |  |
| Health education | | | |
| Non-RBA  RBA | 54 (17.4) / 257  99 (31.2) / 218 | 1.00  2.16 (1.48, 3.15) | 1.00  2.14 (1.44, 3.17) |
| *P value* |  | *<0.001* | *<0.001* |
|  |  |  |  |
| Family planning | | | |
| Non-RBA  RBA | 111 (35.7) / 200  177 (55.8) / 140 | 1.00  2.28 (1.65, 3.14) | 1.00  2.25 (1.61, 3.14) |
| *P value* |  | *<0.001* | *<0.001* |
|  |  |  |  |
| Vaccination | | | |
| Non-RBA  RBA | 98 (31.5) / 213  170 (53.6) / 147 | 1.00  2.51 (1.82, 3.48) | 1.00  2.53 (1.80, 3.55) |
| *P value* |  | *<0.001* | *<0.001* |
|  |  |  |  |
| Antenatal and postnatal check-up | | | |
| Non-RBA  RBA | 131 (42.1) / 180  188 (59.3) / 129 | 1.00  2.00 (1.46, 2.75) | 1.00  2.00 (1.44, 2.78) |
| *P value* |  | *<0.001* | *<0.001* |
|  |  |  |  |
| TB treatment | | | |
| Non-RBA  RBA | 72 (23.2) / 239  148 (46.7) / 169 | 1.00  2.91 (2.06, 4.10) | 1.00  2.90 (2.03, 4.15) |
| *P value* |  | *<0.001* | *<0.001* |
|  |  |  |  |
| Treatment for diarrhoea | | | |
| Non-RBA  RBA | 196 (63.0) / 115  246 (77.6) / 71 | 1.00  2.03 (1.43, 2.89) | 1.00  2.15 (1.49, 3.10) |
| *P value* |  | *<0.001* | *<0.001* |
|  |  |  |  |
| Treatment for pneumonia | | | |
| Non-RBA  RBA | 152 (48.9) / 159  215 (67.8) / 102 | 1.00  2.20 (1.59, 3.05) | 1.00  2.35 (1.67, 3.32) |
| *P value* |  | *<0.001* | *<0.001* |
|  |  |  |  |
| National program services | | | |
| Non-RBA  RBA | 24 ( 7.7) / 287  54 (17.0) / 263 | 1.00  2.46 (1.48, 4.09) | 1.00  2.33 (1.38, 3.96) |
| *P value* |  | *0.001* | *0.002* |
|  |  |  |  |

^1^ For caste, gender, education, food sufficiency, marital status, birth in last 3 years, number of children in household

**Table 10c:** Odds ratio (95% confidence interval) for response of health worker towards patient amongst all 628 participants

|  | N (%) yes / no | Unadjusted | Multiply adjusted^1^ |
| --- | --- | --- | --- |
|  |  |  |  |
| Response of health worker (satisfactory vs. not) | | | |
| Non-RBA  RBA | 279 (91.5) / 26  306 (97.5) / 8 | 1.00  3.56 (1.59, 8.00) | 1.00  2.89 (1.25, 6.70) |
| *P value* |  | *0.002* | *0.01* |
|  |  |  |  |

^1^ For caste, gender, education, food sufficiency, marital status, birth in last 3 years, number of children in household

**Table 10d:** Odds ratio (95% confidence interval) for satisfaction with health institute services amongst all 628 participants

|  | N (%) yes / no | Unadjusted | Multiply adjusted^1^ |
| --- | --- | --- | --- |
|  |  |  |  |
| Satisfaction (satisfactory vs. not) | | | |
| Non-RBA  RBA | 227 (74.2) / 79  257 (81.6) / 58 | 1.00  1.54 (1.05, 2.26) | 1.00  1.69 (1.13, 2.53) |
| *P value* |  | *0.03* | *0.01* |
|  |  |  |  |

^1^ For caste, gender, education, food sufficiency, marital status, birth in last 3 years, number of children in household

**Table 10e:** Odds ratio (95% confidence interval) for satisfaction with Traditional Healer amongst all 628 participants

|  | N (%) yes / no | Unadjusted | Multiply adjusted^1^ |
| --- | --- | --- | --- |
|  |  |  |  |
| Satisfaction (satisfactory vs. not) | | | |
| Non-RBA  RBA | 190 (61.5) / 119  158 (50.3) / 156 | 1.00  0.63 (0.46, 0.87) | 1.00  0.63 (0.45, 0.87) |
| *P value* |  | *0.01* | *0.01* |
|  |  |  |  |

^1^ For caste, gender, education, food sufficiency, marital status, birth in last 3 years, number of children in household

**Table 10f:** **Odds ratio (95% confidence interval) for use and knowledge of health institute services amongst all 628 participants**

|  | N (%) yes / no | Unadjusted | Multiply adjusted^1^ |
| --- | --- | --- | --- |
|  |  |  |  |
| Health workers regularly stay in HI (Agree vs. disagree/don’t know) | | | |
| Non-RBA  RBA | 256 (82.3) / 55  291 (91.8) / 26 | 1.00  2.40 (1.46, 3.95) | 1.00  1.97 (1.18, 3.30) |
| *P value* |  | *0.001* | *0.01* |
|  |  |  |  |
| Going to HI is a waste of money (Disagree vs. agree/don’t know) | | | |
| Non-RBA  RBA | 297 (95.8) / 13  309 (97.5) / 8 | 1.00  1.69 (0.69, 4.14) | 1.00  1.70 (0.67, 4.31) |
| *P value* |  | *0.25* | *0.26* |
|  |  |  |  |
| When any health problem going to HI is best option (Agree vs. disagree/don’t know) | | | |
| Non-RBA  RBA | 299 (96.1) / 12  309 (97.8) / 7 | 1.00  1.77 (0.69, 4.56) | 1.00  1.89 (0.71, 5.04) |
| *P value* |  | *0.24* | *0.20* |
|  |  |  |  |
| Like the response given by health worker (Agree vs. disagree/don’t know) | | | |
| Non-RBA  RBA | 289 (92.9) / 22  311 (98.1) / 6 | 1.00  3.95 (1.58, 9.87) | 1.00  3.46 (1.35, 8.92) |
| *P value* |  | *0.003* | *0.01* |
|  |  |  |  |
| FCHVs are helpful (Agree vs. disagree/don’t know) | | | |
| Non-RBA  RBA | 288 (92.6) / 23  312 (98.4) / 5 | 1.00  4.98 (1.87, 13.28) | 1.00  3.88 (1.42, 10.60) |
| *P value* |  | *0.001* | *0.01* |
|  |  |  |  |
| TB are fully trained (Agree vs. disagree/don’t know) | | | |
| Non-RBA  RBA | 145 (47.1) / 163  207 (66.1) / 106 | 1.00  2.20 (1.59, 3.03) | 1.00  2.22 (1.59, 3.12) |
| *P value* |  | *<0.001* | *<0.001* |
|  |  |  |  |
| TB are very helpful (Agree vs. disagree/don’t know) | | | |
| Non-RBA  RBA | 191 (62.0) / 117  222 (71.2) / 90 | 1.00  1.51 (1.08, 2.11) | 1.00  1.47 (1.03, 2.08) |
| *P value* |  | *0.02* | *0.03* |
|  |  |  |  |

^1^ For caste, gender, education, food sufficiency, marital status, birth in last 3 years, number of children in household

| *P value* |  | *0.02* | *0.004* | *0.37* |
| --- | --- | --- | --- | --- |
|  |  |  |  |  |
